# A Genome-Wide Integrative Genomic Study Localizes Genetic Factors Influencing Antibodies against Epstein-Barr Virus Nuclear Antigen 1 (EBNA-1)

**DOI:** 10.1371/journal.pgen.1003147

**Published:** 2013-01-10

**Authors:** Rohina Rubicz, Robert Yolken, Eugene Drigalenko, Melanie A. Carless, Thomas D. Dyer, Lara Bauman, Phillip E. Melton, Jack W. Kent, John B. Harley, Joanne E. Curran, Matthew P. Johnson, Shelley A. Cole, Laura Almasy, Eric K. Moses, Nikhil V. Dhurandhar, Ellen Kraig, John Blangero, Charles T. Leach, Harald H. H. Göring

**Affiliations:** 1 Department of Genetics, Texas Biomedical Research Institute, San Antonio, Texas, United States of America; 2 Stanley Division of Developmental Neurovirology, Johns Hopkins University School of Medicine, Baltimore, Maryland, United States of America; 3 Centre for Genetic Epidemiology and Biostatistics, The University of Western Australia, Perth, Australia; 4 Division of Rheumatology, Cincinnati Children's Hospital Medical Center, Cincinnati, Ohio, United States of America; 5 Infections and Obesity Laboratory, Pennington Biomedical Research Center, Louisiana State University System, Baton Rouge, Louisiana, United States of America; 6 Department of Cellular and Structural Biology, University of Texas Health Science Center at San Antonio, San Antonio, Texas, United States of America; 7 Department of Pediatrics, University of Texas Health Science Center at San Antonio, San Antonio, Texas, United States of America; Georgia Institute of Technology, United States of America

## Abstract

Infection with Epstein-Barr virus (EBV) is highly prevalent worldwide, and it has been associated with infectious mononucleosis and severe diseases including Burkitt lymphoma, Hodgkin lymphoma, nasopharyngeal lymphoma, and lymphoproliferative disorders. Although EBV has been the focus of extensive research, much still remains unknown concerning what makes some individuals more sensitive to infection and to adverse outcomes as a result of infection. Here we use an integrative genomics approach in order to localize genetic factors influencing levels of Epstein Barr virus (EBV) nuclear antigen-1 (EBNA-1) IgG antibodies, as a measure of history of infection with this pathogen, in large Mexican American families. Genome-wide evidence of both significant linkage and association was obtained on chromosome 6 in the human leukocyte antigen (HLA) region and replicated in an independent Mexican American sample of large families (minimum *p*-value in combined analysis of both datasets is 1.4×10^−15^ for SNPs rs477515 and rs2516049). Conditional association analyses indicate the presence of at least two separate loci within MHC class II, and along with lymphocyte expression data suggest genes *HLA-DRB1* and *HLA-DQB1* as the best candidates. The association signals are specific to EBV and are not found with IgG antibodies to 12 other pathogens examined, and therefore do not simply reveal a general HLA effect. We investigated whether SNPs significantly associated with diseases in which EBV is known or suspected to play a role (namely nasopharyngeal lymphoma, Hodgkin lymphoma, systemic lupus erythematosus, and multiple sclerosis) also show evidence of associated with EBNA-1 antibody levels, finding an overlap only for the HLA locus, but none elsewhere in the genome. The significance of this work is that a major locus related to EBV infection has been identified, which may ultimately reveal the underlying mechanisms by which the immune system regulates infection with this pathogen.

## Author Summary

Many factors influence individual differences in susceptibility to infectious disease, including genetic factors of the host. Here we use several genome-wide investigative tools (linkage, association, joint linkage and association, and the analysis of gene expression data) to search for host genetic factors influencing Epstein-Barr virus (EBV) infection. EBV is a human herpes virus that infects up to 90% of adults worldwide, infection with which has been associated with severe complications including malignancies and autoimmune disorders. In a sample of >1,300 Mexican American family members, we found significant evidence of association of anti–EBV antibody levels with loci on chromosome 6 in the human leukocyte antigen region, which contains genes related to immune function. The top two independent loci in this region were *HLA-DRB1* and *HLA-DQB1*, both of which are involved in the presentation of foreign antigens to T cells. This finding was specific to EBV and not to 12 other pathogens we examined. We also report an overlap of genetic factors influencing both EBV antibody level and EBV–related cancers and autoimmune disorders. This work demonstrates the presence of EBV susceptibility loci and provides impetus for further investigation to better understand the underlying mechanisms related to differences in disease progression among individuals infected with this pathogen.

## Introduction

Epstein-Barr virus (EBV) belongs to the herpes virus family, which consists of double-stranded DNA viruses, composed of a DNA core surrounded by a nucleocapsid and a tegument, with relatively large genomes (100–200 genes). There are currently eight known human herpesviruses, which include herpes simplex virus type I, herpes simplex virus type II, varicella-zoster virus, EBV, cytomegalovirus, human herpesvirus 6, human herpesvirus 7, and Kaposi sarcoma herpesvirus. Infection with EBV is common, with over 90% of the world's adult population estimated to be infected [1]. EBV is thought to be typically transmitted through contact with saliva, infecting B lymphocytes and epithelial cells of the oropharynx [2]. The virus is shed consistently into saliva during primary infection, but intermittent shedding can continue for years afterwards. Following initial infection, EBV establishes a dormant, lifelong infection (mainly in memory B cells) and retains the potential to reactivate [3], [4]. In developing countries primary EBV infection usually occurs during infancy or early childhood, and is typically without clinical symptoms or presents as a mild febrile illness. In more affluent countries infection in childhood is still common, but for approximately one third of individuals primary infection occurs during adolescence or early adulthood and is associated with a higher risk of infectious mononucleosis [5]. EBV infection has also been associated with some malignant conditions including Burkitt lymphoma, nasopharyngeal carcinoma, some gastric cancers, and Hodgkin lymphoma [6]–[11].

EBV has been studied extensively. It was the first human tumor virus identified, and its viral genome was the first to be fully described [12]–[13]. While genetic risk factors have been reported for particular EBV-infected subpopulations (e.g., individuals with Hodgkin lymphoma, infectious mononucleosis, and multiple sclerosis [14]–[19]), there is still much that remains unknown about inter-individual differences in antibody response to EBV exposure and potential adverse outcomes related to infection with this pathogen. Here we have quantified antibody titer to the Epstein-Barr virus nuclear antigen 1 (anti-EBNA-1), which reflects history of infection with this pathogen, in a randomly ascertained sample (i.e., one that is not enriched for a particular phenotype) of Mexican Americans and used genome-wide linkage and association analyses and expression profiling on lymphocytes to identify underlying genetic loci.

## Results

### Seroprevalence

**Figure 1:**
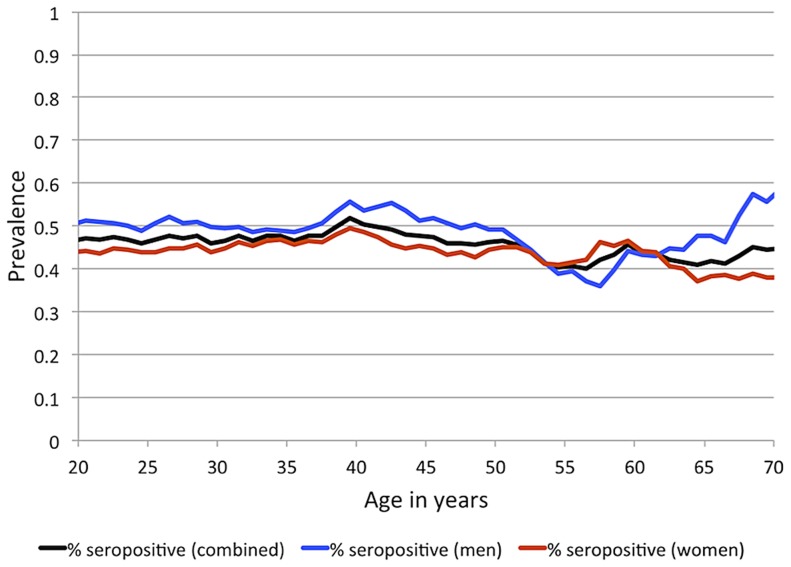
EBV seroprevalence, based on measurement of anti-EBNA-1 antibodies, by sex and age for SAFHS. Sliding 15-year age windows are used to smooth the curves, and age shown is the midpoint of each age interval.

IgG antibodies to EBNA-1 were quantified in plasma samples from 1,367 randomly ascertained and naturally infected Mexican American participants of the San Antonio Family Heart Study (SAFHS), which represent 63 families, many of which are large genealogies (Table S1). While seropositivity to infection with EBV is sometimes measured using IgG antibodies against the viral capsid antigen (VCA), here we refer to EBV seropositivity as based on measurement of anti-EBNA-1 antibodies, which are produced during latent EBV infection. In this study, 48% of individuals are categorized as seropositive for anti-EBNA-1 antibodies, 24% have intermediate (seroindeterminate) levels, and 28% are seronegative. EBNA-1 seroprevalence is similar in men and women and does not change substantially with age within the examined age range of 16–94 years (Figure 1), indicating that most subjects underwent primary infection before adulthood. Given the prevalence of EBV infection and mode of transmission, we assume that essentially all individuals have been exposed to this pathogen multiple times during their lifetime, and therefore seronegative status should be informative in that those individuals failed to mount an antibody-mediated immune response to EBNA-1 (or mounted only a response so weak as to lead to undetectable antibody levels).

### Heritability analysis

While the measured antibody assay generates a quantitative read-out of antibody levels, conceptually the antibody-mediated immune response may be viewed as a yes/no trait, in which the immune system did or did not mount an antibody response after exposure, and thus it may also be justifiable to discretize the assay results. We have performed all analyses presented below on both the quantitative antibody phenotype and the dichotomized serostatus phenotype. For clarity, in the text we pay more attention to the results with the quantitative phenotype. In addition, we view the quantitative trait as more informative and the statistical results to be more reliable, partly because the cut-off levels for discretization are somewhat arbitrary. However, the results for both traits are presented in the Figures and Tables, and the findings are highly consistent with one another.

Heritability rates of EBNA-1 serological phenotypes were estimated using a variance components approach, and shared environment was accounted for by using a “household” random effects model [20]. EBNA-1 serological measures are significantly heritable, at 43% (p≪10^−9^) and 68% (p≪10^−9^) for the quantitative antibody titer and discrete serostatus traits, respectively (Table 1), indicating that host-genetic factors are important determinants of immune response (as we have previously found to be the case for other pathogens [21]). However, household effects, which are one measure of shared environmental exposures and which were based on reported co-habitation at the time of blood draw for serological assaying, are not significant in this sample. Given the high prevalence of this pathogen, this observation may indicate that an individual is just as likely to be infected by someone who does not share the same residence as they are by someone who does.

**Table 1:** Heritability estimates of EBNA-1 quantitative antibody and discrete serostatus traits, with and without household effects.

Trait	Sample size	Heritability without household effects (*p*-value)	Heritability with household effects (*p*-value)	Household effects (*p*-value)
Quantitative	1367	42.8% (2.9×10^−22^)	42.8% (2.4×10^−22^)	3.8% (0.210)
Discrete^a^	1047	68.5% (4.0×10^−16^)	68.7% (3.8×10^−16^)	9.8% (0.179)

a Intermediate antibody titers were coded as unknown (indeterminate), thereby reducing the number of individuals included in analyses of the dichotomous serostatus trait.

### Linkage and association analyses

**Figure 2:**
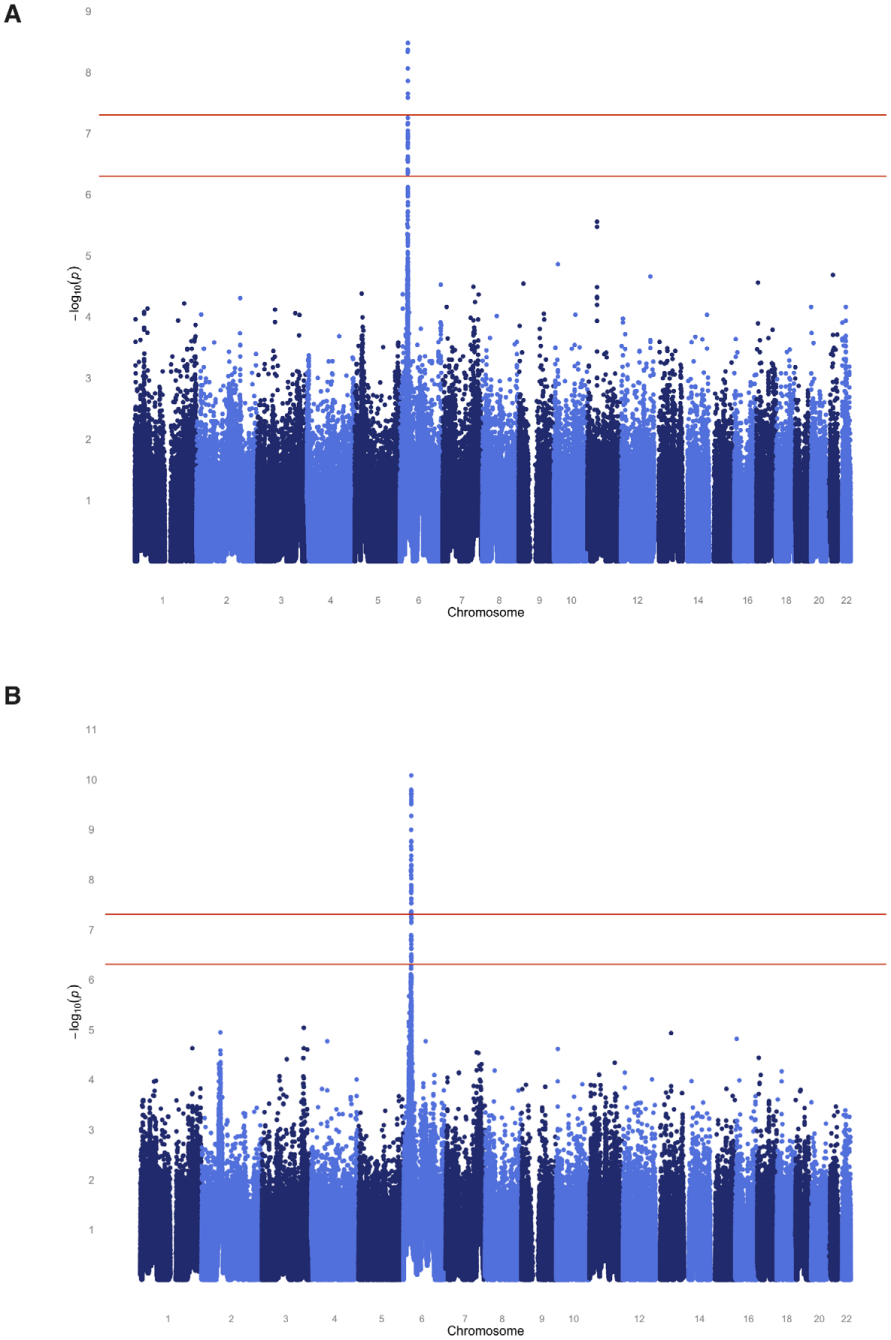
Genome-wide joint linkage and association analysis results for EBNA-1 antibody traits for SAFHS. (A) Quantitative antibody titer. (B) Discrete serostatus.

To localize any underlying genetic factors, we performed a variety of genome-wide analyses using nearly 1 million available SNPs. The SAFHS consists of extended families that provide information on linkage as well as association. We therefore performed joint analysis of linkage and association, thereby using both information sources available in families in order to localize the responsible loci. After Bonferroni correction for the number of SNPs analyzed, multiple genome-wide significant SNPs (most significant *p*-values of 3.3×10^−9^ and 8.3×10^−11^ for the quantitative and dichotomous serological trait, respectively) were found in the human leukocyte antigen (HLA) region on chromosome 6 (Figure 2, Table S2). The HLA region is also implicated by linkage analysis itself, with maximum LOD scores of 1.27 and 3.05 for the quantitative and discrete trait, respectively (Figure S1). No genome-wide significant evidence of linkage and/or association was found elsewhere in the genome.

Having identified the HLA region as a major locus, we performed association analysis conditional on linkage, in order to separate out the association signal from individual SNPs (see Methods section for more detail). The most significant association *p*-value for the quantitative trait occurs with SNPs rs477515 and rs2516049 (*p* = 1.4×10^−8^) (Table 2), two SNPs that are in complete linkage disequilibrium (LD) with each other and are located within the *HLA-DRB1* gene in the HLA class II region. The most significant result for the discrete trait is for SNP rs9268832 in *HLA-DRB9* (*p* = 2.2×10^−9^). In all, 5 and 19 SNPs reach genome-wide significance for the quantitative and discrete EBNA-1 traits, respectively, and all are located in the HLA region of chromosome 6. As shown by the quantile-quantile plot, the HLA region accounts for the entirety of the deviation from the diagonal line of expected p-values under the null hypothesis, and there was no evidence of any inflation in p-values once the HLA region was excluded from the plot (Figure S2).

**Table 2:** Association analysis given linkage.

SNP	Location on chrom 6 (bp)	Nearest gene	Minor allele	Minor allele frequency	This study (SAFHS)		Replicate (SAFDGS)		Combined (SAFHS+SAFDGS)	
					Quantitative *p*-value (ß_SNP_)	Discrete *p*-value (ß_SNP_)^a^	Quantitative *p*-value (ß_SNP_)	Discrete *p*-value (ß_SNP_)^a^	Quantitative *p*-value (ß_SNP_)	Discrete *p*-value (ß_SNP_)^a^
rs3130048	31721718	*BAT3*	C	0.138	**1.89×10^−8^(−0.35)**	6.17×10^−8^(0.51)	0.010(−0.24)	0.052(0.29)	**1.98×10^−9^(−0.32)**	**9.24×10^−8^(0.41)**
rs652888	31959213	*EHMT2*	C	0.096	6.96×10^−7^(−0.36)	**2.82×10^−8^(0.60)**	0.005(−0.30)	0.009(0.48)	**3.46×10^−8^(−0.34)**	**2.26×10^−9^(0.55)**
rs204999	32217957	*PRRT1*	G	0.180	**3.61×10^−8^(−0.30)**	**4.25×10^−9^(0.49)**	1.11×10^−5^(−0.38)	9.39×10^−6^(0.58)	**2.89×10^−12^(−0.33)**	**1.64×10^−12^(0.49)**
rs4248166	32474399	*BTNL2*	C	0.254	**1.75×10^−8^(0.27)**	**6.78×10^−9^(−0.43)**	0.002(0.23)	0.075(−0.21)	**1.05×10^−10^(0.26)**	**1.04×10^−9^(−0.38)**
rs2294884	32475237	*BTNL2*	C	0.261	2.45×10^−7^(0.24)	**4.82×10^−8^(−0.40)**	0.003(0.22)	0.131(−0.18)	**1.80×10^−9^(0.23)**	**1.72×10^−8^(−0.34)**
rs2294882	32475493	*BTNL2*	G	0.280	1.12×10^−7^(0.24)	**4.58×10^−9^(−0.42)**	0.003(0.22)	0.109(−0.19)	**9.42×10^−10^(0.24)**	**1.70×10^−9^(−0.36)**
rs2294881	32475582	*BTNL2*	G	0.280	1.12×10^−7^(0.24)	**4.58×10^−9^(−0.42)**	0.003(0.22)	0.109(−0.19)	**9.42×10^−10^(0.24)**	**1.70×10^−9^(−0.36)**
rs28362680	32478794	*BTNL2*	T	0.235	5.53×10^−7^(0.24)	**8.37×10^−9^(−0.46)**	0.009(0.20)	0.185(−0.16)	**1.64×10^−8^(0.23)**	**7.52×10^−9^(−0.37)**
rs28362683	32480941	*BTNL2*	T	0.220	6.82×10^−7^(0.25)	**5.02×10^−8^(−0.44)**	0.011(0.19)	0.209(−0.15)	**2.56×10^−8^(0.23)**	**4.05×10^−8^(−0.36)**
rs10947261	32481210	*BTNL2*	T	0.233	4.54×10^−7^(0.25)	**7.40×10^−9^(−0.46)**	0.009(0.20)	0.193(−0.16)	**1.31×10^−8^(0.23)**	**7.34×10^−9^(−0.38)**
rs10947262	32481290	*BTNL2*	T	0.231	1.10×10^−6^(0.24)	**1.46×10^−8^(−0.45)**	0.009(0.20)	0.185(−0.16)	**3.05×10^−8^(0.22)**	**1.23×10^−8^(−0.37)**
rs7192	32519624	*HLA-DRA*	T	0.389	7.81×10^−7^(0.21)	**8.14×10^−9^(−0.40)**	0.010(0.16)	0.163(−0.14)	**3.44×10^−8^(0.20)**	**1.16×10^−8^(−0.32)**
rs2239803	32519811	*HLA-DRA*	G	0.453	2.06×10^−6^(0.20)	**5.01×10^−8^(−0.36)**	0.016(0.17)	0.240(−0.11)	**1.13×10^−7^(0.19)**	**5.75×10^−8^(−0.30)**
rs7194	32520458	*HLA-DRA*	G	0.391	5.24×10^−7^(0.22)	**5.20×10^−9^(−0.40)**	0.010(0.16)	0.163(−0.14)	**2.39×10^−8^(0.20)**	**7.78×10^−9^(−0.33)**
rs7195	32520517	*HLA-DRA*	A	0.391	5.24×10^−7^(0.22)	**5.20×10^−9^(−0.40)**	0.010(0.16)	0.163(−0.14)	**2.39×10^−8^(0.20)**	**7.78×10^−9^(−0.33)**
rs2213586	32521072	*HLA-DRA*	T	0.391	5.24×10^−7^(0.22)	**5.20×10^−9^(−0.40)**	0.010(0.16)	0.163(−0.14)	**2.39×10^−8^(0.20)**	**7.78×10^−9^(−0.33)**
rs2213585	32521128	*HLA-DRA*	C	0.391	5.24×10^−7^(0.22)	**5.20×10^−9^(−0.40)**	0.010(0.16)	0.163(−0.14)	**2.39×10^−8^(0.20)**	**7.78×10^−9^(−0.33)**
rs2227139	32521437	*HLA-DRA*	C	0.391	5.24×10^−7^(0.22)	**5.20×10^−9^(−0.40)**	0.010(0.16)	0.163(−0.14)	**2.39×10^−8^(0.20)**	**7.78×10^−9^(−0.33)**
rs7754768	32528157	*HLA-DRA*	C	0.402	5.06×10^−7^(0.21)	**5.85×10^−9^(−0.39)**	0.010(0.16)	0.137(−0.15)	**2.13×10^−8^(0.20)**	**8.29×10^−9^(−0.32)**
rs9268832	32535767	*HLA-DRB9*	T	0.385	9.63×10^−8^(0.23)	**2.16×10^−9^(−0.41)**	0.029(0.14)	0.197(−0.14)	**1.73×10^−8^(0.20)**	**5.41×10^−9^(−0.33)**
rs477515	32677669	*HLA-DRB1*	T	0.320	**1.36×10^−8^(−0.26)**	8.72×10^−8^(0.37)	**4.07×10^−6^(−0.32)**	**8.76×10^−7^(0.55)**	**3.07×10^−13^(−0.28)**	**3.91×10^−12^(0.39)**
rs2516049	32678378	*HLA-DRB1*	G	0.320	**1.36×10^−8^(−0.26)**	8.72×10^−8^(0.37)	**4.07×10^−6^(−0.32)**	**8.76×10^−7^(0.55)**	**3.07×10^−13^(−0.28)**	**3.91×10^−12^(0.39)**

Shown are all SNPs yielding genome-wide significant *p*-values with either the quantitative and/or the qualitative antibody phenotype in the SAFHS. The regression coefficients refer to the estimated change in the phenotype for each dose of the rarer SNP allele. For the SAFHS all significant genome-wide results (*p*≤5.29×10^−8^) are presented in bold lettering. After correcting for multiple testing during replication in the SADGS (we tested the entire HLA region, with 5689 available SNPs: *p*≤0.05/5689≈8.79×10^−6^), there are two significant SNPs in the replication sample. When using the combined sample of both studies (SAFHS+SAFDGS), all SNPs originally significant in the SAFHS discovery sample are highly significant.

a Since we used a liability threshold model for analysis of the dichotomous trait (see methods section), the direction of effect on EBNA-1 discrete serostatus is opposite of the sign of the regression coefficient, but is in the same direction as the regression coefficient for the quantitative trait (e.g. for SNP rs204999, the minor allele is associated with a decrease in EBNA-1 antibody level and seronegativity).

### Replication

To confirm our findings, we generated anti-EBNA-1 antibody measurements (using the same assay) in plasma samples from 589 participants (representing 39, mainly extended families [Table S1]) in a separate Mexican American family cohort from San Antonio, the San Antonio Family Diabetes/Gall Bladder Study (SAFDGS) [22]. EBNA-1 seroprevalence in this cohort was estimated to be somewhat higher at 85%. Heritability estimates are 37% (p = 1.0×10^−6^) and 42% (p = 1.9×10^−3^) for the quantitative and discrete traits, respectively, nearly identical to the prior estimates from the SAFHS. Linkage analysis yielded LOD scores of 3.37 and 2.22 in the HLA region on chromosome 6 for the quantitative and dichotomous traits, respectively (Figure S3). Also, genome-wide joint linkage and association analysis points to significant results only for chromosome 6, and not elsewhere in the genome (Figure S4). We then performed association analysis conditional on linkage of all SNPs from the extended HLA region, and after Bonferroni correction for the number of SNPs analyzed we obtained significant evidence of association with the same two SNPs (rs477515/rs2516049) (most significant *p*-values of 4.1×10^−6^ and 8.8×10^−7^ for the quantitative and discrete traits, respectively) (Table 2).

**Figure 3:**
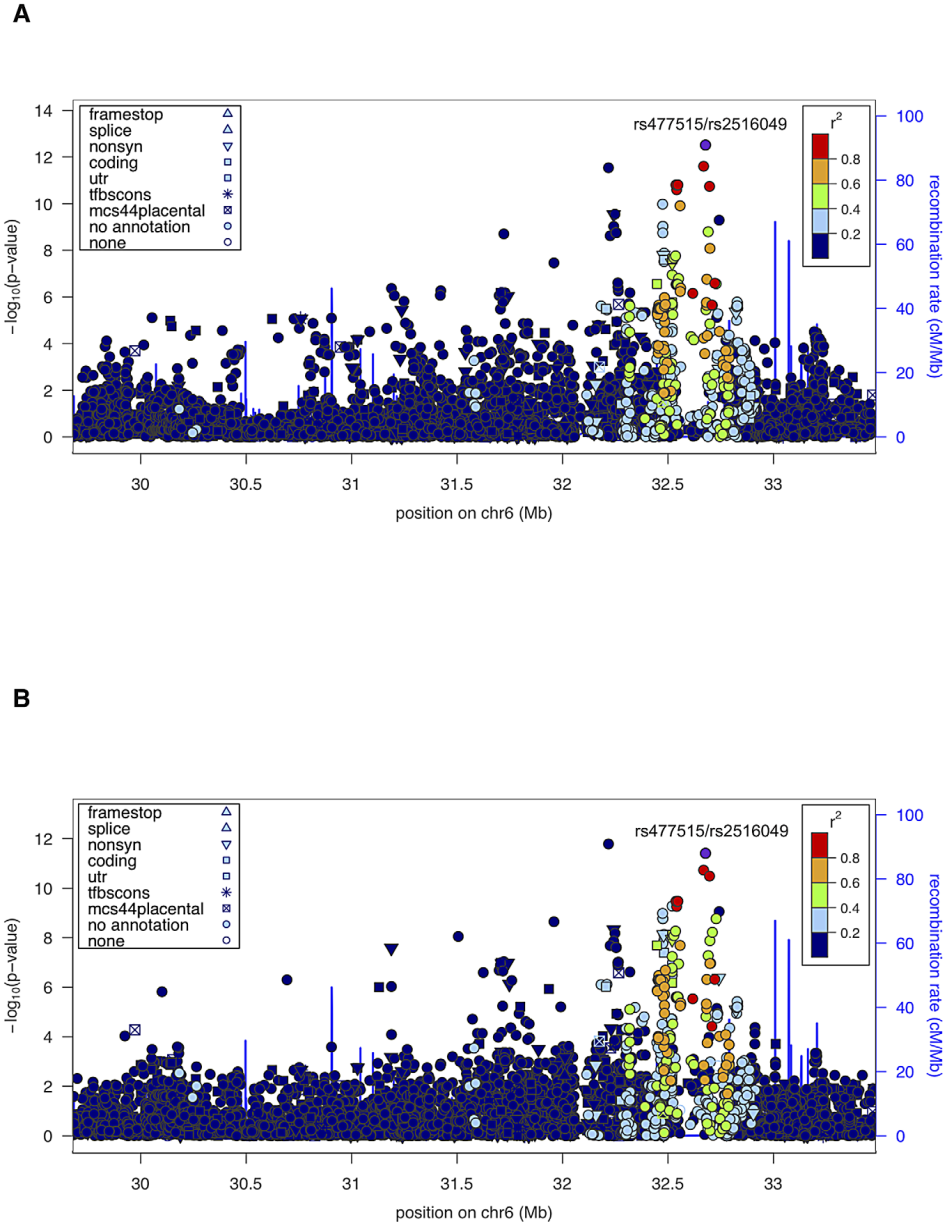
Association analysis results (conditional on linkage) of extended HLA region for the combined sample (SAFHS+SAFDGS). The LD pattern was estimated based on SNP genotypes from study participants. SNPs in red are highly correlated with the top SNP associated with the EBNA-1 quantitative trait (rs477515/rs2516049). (A) Quantitative antibody titer. (B) Discrete serostatus.

Given the unambiguous replication of the HLA locus, including evidence of linkage and association in the extended HLA region in both datasets, we also performed joint analysis of both pedigree cohorts (SAFHS+SAFDGS) using all available SNPs in the extended HLA region, in order to use all available information for identification and prioritization of the most candidate SNPs within this region. The minimum *p*-values for association analysis conditional on linkage for the combined dataset were even more significant and occurred at the very SNPs that gave the most significant results for each dataset alone (*p* = 3.1×10^−13^ and *p* = 3.9×10^−12^ for SNPs rs477515/rs2516049 for the quantitative and discrete traits, respectively) (Figure 3, Table 2).

### Conditional association analyses

The HLA region is highly complex and exhibits considerable and long-range LD. In order to determine whether a single or multiple haplotype block harbors genetic variants influencing the serological EBNA-1 phenotypes, we performed several rounds of conditional association analysis within the extended HLA region using the combined sample of both studies. We first conditioned on the most significant SNP for the quantitative trait (rs477515/rs2516049) and identified one additional significant SNP (rs2854275, located within the *HLA-DQB1* gene in the MHC class II region) that was independently associated with EBNA-1 at a genome-wide level of significance (Table 3, Figure S5). After conditioning on both independent SNPs (rs477515/rs2516049 and rs2854275), no additional SNPs were significant for the quantitative antibody trait. This suggests that at least two haplotype blocks harbor variants influencing EBNA-1 seroreactivity. The pattern of LD among SNPs giving genome-wide significant association evidence with either EBNA-1 quantitative or dichotomous EBNA-1 trait is shown in Figure S6.

**Table 3:** Association analysis, given linkage, conditional on the top SNP.

SNP	Location on chrom 6 (bp)	Nearest gene	Minor allele	Minor allele frequency	*p*-value (ß_SNP_)^a^	
					Quantitative trait	Discrete trait
rs2233971	31188302	*C6orf15*	A	0.003	0.291 (0.05)	**1.95×10^−8^ (−0.33)**
rs2233956	31189184	*C6orf15*	G	0.067	**7.35×10^−9^ (−0.40)**	4.93×10^−6^ (0.49)
rs3130557	31202682	*PSORS1C1*	T	0.029	**4.38×10^−8^(−0.52)**	9.60×10^−5^ (0.57)
rs2854008	31420517	*HLA-B*	T	0.195	**3.38×10^−8^(−0.24)**	2.53×10^−4^ (0.26)
rs2507997	31422760	*HLA-B*	C	0.190	**2.07×10^−8^(−0.24)**	2.11×10^−4^ (0.26)
rs3093970	31505856	*SDK1*	A	0.003	0.233 (0.05)	**4.98×10^−9^** **(−0.34)**
rs3130070	31699787	*BAT2*	G	0.083	**2.57×10^−8^ (−0.36)**	**5.02×10^−9^ (0.56)**
rs3130623	31705679	*BAT2*	T	0.089	1.02×10^−7^ (−0.34)	**2.49×10^−9^ (0.56)**
rs3130626	31706468	*BAT2*	G	0.084	**2.22×10^−8^ (−0.36)**	**2.71×10^−9^ (0.57)**
rs2736157	31708799	*BAT2*	C	0.084	**2.22×10^−8^ (−0.36)**	**2.71×10^−9^ (0.57)**
rs3115663	31709822	*BAT2*	G	0.088	**2.22×10^−8^ (−0.36)**	**2.66×10^−9^ (0.57)**
rs9267522	31711749	*BAT2*	G	0.088	**2.22×10^−8^ (−0.36)**	**2.71×10^−9^ (0.57)**
rs10885	31712570	*BAT2*	T	0.083	**3.42×10^−8^ (−0.35)**	**2.89×10^−9^ (0.56)**
rs3130628	31717251	*BAT3*	C	0.088	**5.02×10^−8^ (−0.35)**	**5.17×10^−9^ (0.55)**
rs3130048	31721718	*BAT3*	C	0.138	**2.39×10^−10^ (−0.33)**	**1.55×10^−8^ (0.44)**
rs3117583	31727555	*BAT3*	C	0.088	**2.22×10^−8^ (−0.36)**	**2.71×10^−9^ (0.57)**
rs3130618	31740113	*BAT4*	A	0.088	**3.08×10^−8^ (−0.36)**	**2.71×10^−9^ (0.57)**
rs652888	31959213	*EHMT2*	C	0.096	**1.84×10^−9^ (−0.36)**	**3.22×10^−10^ (0.58)**
rs204999	32217957	*PRRT1*	G	0.180	**9.77×10^−10^ (−0.28)**	**6.24×10^−10^ (0.43)**
rs9267947	32319196	*NOTCH4*	G	0.391	9.24×10^−8^ (−0.19)	**4.47×10^−8^ (0.31)**
rs9273327	32731201	*HLA-DQB1*	C	0.069	**7.03×10^−10^ (−0.41)**	**7.38×10^−10^ (0.60)**
rs2854275	32736406	*HLA-DQB1*	T	0.064	**2.33×10^−10^ (−0.45)**	**6.50×10^−10^ (0.66)**

The top SNP, rs477515/rs2516049, was determined based on analysis of the quantitative antibody trait in the combined sample (SAFHS and SAFDGS). Only the extended HLA region was analyzed. Shown are the SNPs yielding genome-wide significant *p*-values (*p*≤5.29×10^−8^, shown in bold) with either the quantitative and/or the qualitative antibody phenotype in the combined sample. The regression coefficients refer to the estimated change in the phenotype for each dose of the rarer SNP allele.

a Since we used a liability threshold model for analysis of the dichotomous trait (see methods section), the direction of effect on EBNA-1 discrete serostatus is opposite of the sign of the regression coefficient, but is in the same direction as the regression coefficient for the quantitative trait (e.g. for SNP rs204999, the minor allele is associated with a decrease in EBNA-1 antibody level and seronegativity).

### Expression profile analysis

In order to pinpoint the most likely gene(s) influencing anti-EBNA-1 antibodies, we used an integrative genomics approach based on available expression profiles from 1,243 peripheral blood mononuclear cell (PBMC) samples (collected at the same point in time as the plasma samples used for antibody assays) from SAFHS study participants. Specifically, we examined whether the SNPs that are significantly associated with anti-EBNA-1 antibody status are also significantly associated with expression levels of any nearby gene transcripts (which would suggest that such SNPs are putative *cis*-regulatory variants of these transcripts), and whether those transcript levels in turn are significantly associated with antibody status.

Using the 41 SNPs that were significantly associated with EBNA-1 seroreactivity in the combined SAFHS+SAFDGS sample (with either the quantitative and/or discrete trait, and in the initial and/or subsequent association analysis, i.e. the SNPs included in Table 2 and Table 3), we conducted association analyses on the 150 expressed transcripts from the extended HLA region, yielding 6,750 SNP-transcript pairs. After Bonferroni correction, we observed significant association results (conditional on linkage) for four SNP-transcript pairs (Table S3). SNPs rs204999 and rs10947261 are significantly associated with expression of the housekeeping gene *RPS18* (*p* = 4.8×10^−5^ and *p* = 3.0×10^−4^, respectively), and SNPs rs9273327 and rs2854275 are significantly associated with *PBX2* expression. *PBX2* is a gene involved in B-cell and certain T-cell leukemias. Our results indicate that that these SNPs (or variants in LD with them) may be putative *cis*-acting regulators of these genes. However, the evidence of association is only of moderate strength for any SNP-transcript pair (the *p*-values are significant after Bonferroni correction for the number of transcripts analyzed within the extended HLA region, but not if one were to impose a genome-wide multiple testing correction). In addition, the distance between SNPs and probes is fairly large compared to commonly observed, strong *cis*-acting expression nucleotides. Other SNP-transcript pairs yielded suggestive, but not statistically significant, results after correction for multiple testing.

We subsequently looked at whether any HLA transcripts were significantly correlated with EBNA-1 seroreactivity. However, the expression levels of *RPS18* and *PBX2*, which we had found to be potentially *cis*-regulated by SNPs associated with EBNA-1 antibody phenotypes, were not significantly correlated with the EBNA-1 traits (*p* = 0.17 and *p* = 0.82, for *RPS18* and *PBX2* respectively, for the quantitative trait). Among the other HLA transcripts, the expression level of *HLA-DRB1* is most significantly associated with both anti-EBNA-1 traits (quantitative: *p* = 2.8×10^−5^; discrete: *p* = 3.0×10^−4^) (Table S4). Thus, in conclusion, while our integrative genomic analyses point to potential candidate genes, the evidence obtained does not yield overwhelming support for a particular candidate gene. A potential explanation may be the fact that the relevant differences in HLA function are attributable to alteration in protein sequence or binding affinity rather than gene expression level.

### Examination of other pathogens

As the HLA region is well known to play a role in many aspects of the immune system, it may not be surprising that genetic variants therein appear to influence anti-EBNA-1 antibody levels. To examine whether the identified locus is specific to EBNA-1, or whether it plays a role in determining antibody titers more generally, we assessed whether the EBNA-1-associated SNPs are also significantly associated with antibodies directed at other herpesviruses or other pathogens. IgG **a**ntibody titers had previously been measured on the same plasma samples [23] for 12 other pathogens, including 5 herpesviruses. We focused on the top two independent SNPs associated with the quantitative EBV antibody titer in the SAFHS, and found no evidence of association of SNPs rs477515/rs2516049 or rs2854275 with any of these other pathogens, suggesting that the identified loci are specific to EBV (Table 4) and not some general IgG HLA control mechanism.

**Table 4:** Association (conditional on linkage) results for 12 comparative pathogens.

Pathogen	rs477515/rs2516049 *p-values*: quantitative trait, discrete serostatus trait	rs2854275 *p-values*: quantitative trait, discrete serostatus trait
**EBV**	**3.1×10^−13^, 3.9×10^−12^**	**2.3×10^−10^, 6.5×10^−10^**
CMV	0.89, 0.45	0.99, 0.72
HSV-1	0.86, 0.71	0.03, 0.58
HSV-2	0.23, 0.15	0.73, 0.54
HHV-6	0.19, 0.53	0.06, 0.88
VZV	0.06, 0.22	0.95, 0.31
Ad36	0.54, 0.72	0.69, 0.88
HAV	0.61, 0.53	0.54, 0.62
Influenza A	0.08, 0.09	0.92, 0.50
Influenza B	0.43, 0.33	0.09, 0.28
C. pneumoniae	0.05, 0.11	0.21, 0.99
H. pylorii	0.45, 0.95	0.57, 0.33
T. gondii	0.68, 0.44	0.64, 0.18

Focus is on the independent EBNA-1 associated SNPs rs477515/rs2516049 and rs2854275. There is no significant association of the top EBV SNPs with any of the other pathogens, after applying a Bonferroni correction to account for multiple testing (*p*≤0.05/12≈0.004).

### Relationship to EBV–related cancer

Several types of cancer have been linked to infection with EBV, we therefore looked for evidence of genetic overlap between anti-EBNA-1 antibody traits and published susceptibility loci for two common EBV-related cancers, nasopharyngeal carcinoma (NPC) and Hodgkin lymphoma (HL). A comparison with Burkitt lymphoma was not made, however, as genetic association loci were not available in the published literature. The results for association analysis (conditional on linkage) for EBNA-1 quantitative and discrete traits for NPC-related SNPs are presented in Table 5, and Table 6 presents the results for HL-related SNPs. Two NPC SNPs, rs2860580 and rs28421666, were significantly associated with the EBNA-1 traits, after applying a Bonferroni correction to account for testing 23 SNPs. Both SNPs are located on chromosome 6, in HLA class I and II regions, respectively. Similarly, four HL SNPs that were found to be significantly associated with EBNA-1 trait (rs204000, rs9268542, rs2395185, and rs2858870) were also located in the HLA region. EBNA-1 traits were not significantly associated with cancer-related SNPs that are located outside the HLA region.

**Table 5:** *P*-values for EBNA-1 association, conditional on linkage, analysis for top nasopharyngeal carcinoma SNPs.

SNP	Reference	Chromosome	Base pair location	EBNA-1 quantitative trait: *p*-value for association analysis	EBNA-1 discrete trait: *p*-value for association analysis	Genes of interest
rs2212020	[61]	3	37492466	0.381	0.306	*ITGA9*
rs6774494	[62]	3	170565327	0.225	0.583	*MECOM*
rs2267633	[63]	6	29678820	0.874	0.825	*GABBR1*
rs2076483	[63]	6	29679524	0.874	0.825	*GABBR1*
rs29230	[63]	6	29684372	0.589	0.340	*GABBR1*
rs29232	[63]	6	29719410	0.723	0.241	*GABBR1*
rs3129055	[63]	6	29778240	0.327	0.119	*HLA-F*
rs9258122	[63]	6	29779719	0.327	0.119	*HLA-F*
**rs2860580**	[**62**]	**6**	**30014670**	**1.14×10^−4^**	0.003	***HLA-A***
rs9260734	[63], [64]	6	30040645	0.075	0.084	*HCG9*
rs3869062	[63], [64]	6	30042870	0.649	0.915	*HCG9*
rs5009448	[63]	6	30048467	0.571	0.890	*HCG9*
rs16896923	[63]	6	30108666	0.808	0.802	*HCG9*
rs2894207	[62]	6	31371730	0.483	0.523	*HLA-B/C*
**rs28421666**	[**62**]	**6**	**32700715**	0.009	**0.001**	***HLA-DQ/DR***
rs1412829	[62]	9	22033926	0.290	0.106	*CDKN2A/2B*
rs9418990	[65]	10	135187956	0.503	0.716	*CYP2E1*
rs915906	[65]	10	135193728	0.022	0.115	*CYP2E1*
rs2249695	[65]	10	135202158	0.053	0.188	*CYP2E1*
rs1536826	[65]	10	135207229	0.381	0.550	*CYP2E1*
rs1572072	[62]	13	23025210	0.046	0.012	*TNFRSF19*
rs9510787	[62]	13	23103195	0.896	0.899	*TNFRSF19*
rs927220	[66]	14	67837725	0.846	0.690	*RAD51L1*

Bold = *p*-value significant after Bonferoni correction for multiple testing (0.05/23≈2.17×10^−3^).

**Table 6:** *P*-values for EBNA-1 association, conditional on linkage, analysis for top Hodgkin lymphoma SNPs.

SNP	Reference	Chromosome	Base pair location	EBNA-1 quantitative trait: *p*-value for association analysis	EBNA-1 discrete trait: *p*-value for association analysis	Genes of interest
rs1432295	[67]	2	60920170	0.266	0.681	*REL*
rs2734986	[68]	6	29926547	0.011	0.104	*HLA-A*
rs6904029	[68]	6	30051046	0.043	0.309	*HCG9*
rs2248462	[68]	6	31554775	0.013	0.011	*MICB*
**rs204999**	[**69**]	**6**	**32217957**	**2.89×10^−12^**	**1.64×10^−12^**	***PRRT1***
**rs9268542**	[**69**]	**6**	**32492699**	0.017	**9.66×10^−4^**	***BTNL2, HLA-DRA***
rs6903608	[67]	6	32536263	0.102	0.189	*HLA-DRA*
**rs2395185**	[**68**]	**6**	**32541145**	**2.53×10^−11^**	**5.52×10^−10^**	***HLA-DRA***
**rs2858870**	[**69**]	**6**	**32680229**	**3.52×10^−6^**	**1.52×10^−5^**	***HLA-DRB1, HLA-DQA1***
rs2608053	[67]	8	129145014	0.200	0.882	*PVT1*
rs2019960	[67]	8	129261453	0.120	0.217	*PVT1*
rs501764	[67]	10	8133040	0.657	0.924	*GATA3*
rs485411	[67]	10	8133191	0.706	0.646	*GATA3*

Bold = *p*-value significant after Bonferoni correction for multiple testing (0.05/13≈3.85×10^−3^).

### Relationship to autoimmune diseases

Because EBV infection has been associated with certain autoimmune diseases, in particular systemic lupus erythematosus (SLE) and multiple sclerosis (MS), we examined whether there is evidence for overlap of genetic factors influencing these traits and anti-EBNA-1 antibody traits by investigating whether any of the previously reported SNPs significantly associated with these disorders also show association with EBNA-1 antibody traits. Table 7 provides the results of the association analysis (conditional on linkage) for EBNA-1 quantitative and discrete traits with SLE-associated SNPs taken from the literature, and Table 8 focuses on SNPs associated with MS. After applying a Bonferroni correction for examining 47 SLE-relevant SNPs, rs9268832 and rs9271366 were significantly associated with both EBNA-1 serological traits, and rs3135391 with the discrete EBNA-1 trait. Notably, all three of these SNPs are located in the HLA region on chromosome 6. None of the 41 SLE-associated SNPs outside of the HLA region show any evidence of association to EBNA-1. A comparison with 30 genome-wide significant MS SNPs from published reports yielded similar results, with statistically significant association results, for both EBNA-1 traits with SNP rs9271366, and also for the discrete EBNA-1 serostatus trait with SNPs rs3129860 and rs3135388, all of which are located within the HLA region (Table 8). As with SLE, there was no evidence for association of EBNA-1 with MS-associated SNPs from anywhere else in the genome.

**Table 7:** *P*-values for EBNA-1 association, conditional on linkage, analysis for top systemic lupus erythematosus SNPs.

SNP	Reference	Chromosome	Base pair location	EBNA-1 quantitative trait: *p*-value for association analysis	EBNA-1 discrete trait: *p*-value for association analysis	Genes of interest
rs2476601	[70], [71], [72]	1	114179091	0.625	0.671	*PTPN22*
rs1801274	[71], [73], [74]	1	159746369	0.883	0.716	*FCGR2A*
rs2205960	[70], [75], [76]	1	171458098	0.506	0.773	*OX40L, TNFSF4*
rs3024505	[70]	1	205006527	0.400	0.512	*IL10*
rs13385731	[75]	2	33555394	0.465	0.746	*RASGRP3*
rs1990760	[77]	2	162832297	0.871	0.248	*IFIH1*
rs3024866	[70], [71], [75], [78]–[80]	2	191631086	0.530	0.514	*STAT4*
rs7574865	[70], [71], [75], [78]–[80]	2	191672878	0.391	0.928	*STAT4*
rs3135945	[81], [82]	3	48483970	0.020	0.408	*TREX1*
rs6445975	[70], [71], [80]	3	58345217	0.906	0.789	*PXK*
rs10516487	[70], [80], [83]	4	102970099	0.184	0.859	*BANK1*
rs907715	[84], [85]	4	123754503	0.549	0.149	*IL2/IL21*
rs10036748	[70], [75]	5	150438339	0.804	0.904	*TNIP1*
rs2507987	[86]	6	31451012	0.400	0.523	*HLA-B, MICA*
rs409558	[86]	6	31816126	0.376	0.653	*MSH5*
rs3131379	[70], [71], [78], [79]	6	31829012	2.21×10^−3^	3.47×10^−3^	*MSH5*
rs1270942	[70], [71], [75], [79], [87], [88]	6	32026839	2.21×10^−3^	3.47×10^−3^	*HLA and other genes*
**rs3135391**	[**70**], [71], [78], [**79**]	**6**	**32518965**	2.84×10^−3^	**4.21×10^−4^**	***HLA-DRA***
**rs9268832**	[**86**]	**6**	**32535767**	**9.63×10^−8^**	**2.16×10^−9^**	***HLA-DRB9***
**rs9271366**	[**86**]	**6**	**32694832**	**8.10×10^−4^**	**5.55×10^−5^**	***HLA-DRB1, HLA-DQA1***
rs2071351	[86]	6	33151908	0.587	0.929	*HLA-DPB1*
rs3117213	[86]	6	33172583	0.214	0.735	*HLA-DPB1, HLA-DPB2*
rs11755393	[70]	6	34932614	0.612	0.682	*UHRF1BP1*
rs548234	[71], [75], [79]	6	106674727	0.776	0.876	*ATG5*
rs6568431	[70], [71], [75]	6	106695499	0.468	0.539	*PRDM1, ATG5*
rs2230926	[70], [75], [79], [89]	6	138237759	0.868	0.218	*TNFAIP3*
rs4917014	[75]	7	50276409	0.671	0.509	*IKZF1*
rs10488631	[70], [71], [75], [78]	7	128381419	0.226	0.163	*IRF5*
rs12537284	[70], [71], [79], [90]–[93]	7	128505142	0.322	0.240	*IRF5*
rs6985109	[71], [94]	8	10798995	0.837	0.554	*XKR6*
rs7812879	[75]	8	11377591	0.636	0.614	*BLK*
rs13277113	[70], [75], [78]–[80]	8	11386595	0.777	0.689	*BLK, C8orf13*
rs7829816	[71], [95]	8	57011940	0.909	0.609	*LYN*
rs1913517	[75]	10	49789060	0.738	0.768	*LRRC18*
rs4963128	[70], [71], [80]	11	579564	0.960	0.735	*PHRF1, KIAA1542*
rs507230	[96], [97]	11	35085748	0.075	0.062	*CD44*
rs6590330	[75]	11	127816269	0.154	0.184	*ETS1*
rs10847697	[75]	12	127865338	0.329	0.625	*SLC15A4*
rs1385374	[75]	12	127866647	0.365	0.670	*SLC15A4*
rs7329174	[98]	13	40456110	0.782	0.378	*ELF1*
rs16972959	[99]	16	23808877	0.029	0.0377	*PRKCB*
rs9888739	[70], [71], [78], [79], [100], [101]	16	31220754	0.613	0.491	*ITGAM*
rs2280381	[77]	16	84576134	0.464	0.164	*IRF8*
rs280519	[77]	19	10333933	0.403	0.984	*TYK2*
rs4810485	[102]	20	44181354	0.672	0.865	*CD40*
rs463426	[71], [75]	22	20139185	0.259	0.051	*HIC2*
rs131654	[71], [75]	22	20247190	0.100	0.133	*UBE2L3*

Bold = *p*-value significant after Bonferoni correction for multiple testing (0.05/47≈1.06×10^−3^).

**Table 8:** *P-values* for EBNA-1 association, conditional on linkage, analysis for top multiple sclerosis SNPs.

SNP	Reference	Chromosome	Base pair location	EBV quantitative trait: p-value for association analysis	EBV discrete trait: p-value for association analysis	Genes of interest
rs6604026	[103]	1	93076191	0.224	0.952	*EV1, RPL5*
rs12044852	[104]	1	116889302	0.762	0.474	*CD58*
rs1335532	[103]	1	116902480	0.242	0.077	*CD58*
rs2300747	[104]	1	116905738	0.182	0.093	*CD58*
rs1109670	[105]	2	9167489	0.729	0.517	*DDEF2*
rs651477	[105]	2	119112161	0.630	0.551	*EN1*
rs882300	[104]	2	136692725	0.267	0.079	*CXCR4*
rs908821	[105]	3	142023408	0.812	0.712	*SLC25A36*
rs1841770	[105]	3	149239376	0.027	0.096	*ZIC1*
rs7672826	[105]	4	182636689	0.388	0.731	*MGC458000*
rs6897932	[106]	5	35910332	0.606	0.749	*IL7R*
rs3129934	[107]	6	32444165	0.022	4.54×10^−3^	*HLA-DRB1*
**rs3129860**	[**105**], [**108**]	**6**	**32509057**	1.84×10^−3^	**3.84×10^−5^**	***HLA-DRA***
rs3135338	[109]	6	32509195	0.088	0.013	*HLA-DRA*
**rs3135388**	[**104]**, [**108**]	**6**	**32521029**	1.33×10^−3^	**1.54×10^−4^**	***HLA-DRA, HLA-DRB1***
**rs9271366**	[**103**], [**109**]	**6**	**32694832**	**8.10×10^−4^**	**5.55×10^−5^**	***HLA-DQA1, HLA-DRB1***
rs1529316	[105]	8	3815546	0.221	0.107	*CSMD1*
rs1755289	[105]	9	17928351	0.043	0.141	*SH3GL2*
rs3780792	[110]	9	135825164	0.587	0.984	*VAV2*
rs2104286	[104]	10	6139051	0.774	0.928	*IL2RA*
rs2503875	[110]	10	43134055	0.973	0.812	*intergenic*
rs1800693	[104]	12	6310270	0.199	0.224	*TNFRSF1A*
rs4149584	[104]	12	6312904	0.298	0.183	*TNFRSF1A*
rs1458175	[105]	12	40252128	0.280	0.437	*PDZRN4*
rs703842	[103]	12	56449006	0.450	0.729	*METTL1, CYP27B1*
rs9523762	[105]	13	92129887	0.451	0.439	*GPC5*
rs744166	[109]	17	37767727	0.314	0.407	*STAT3*
rs397020	[104]	20	1153886	0.399	0.278	*C20orf46*
rs6074022	[103]	20	44173603	0.802	0.797	CD40
rs1569723	[103]	20	44175471	0.802	0.797	CD40

Bold = *p*-value significant after Bonferoni correction for multiple testing (0.05/30≈9.09×10^−4^).

## Discussion

In this study, we estimated the seroprevalence rate of EBV infection as 48% seropositive in the study population of 1,367 Mexican American participants from the SAFHS. Our estimate is lower than estimates of EBV prevalence for other adult populations [24], but this study characterized anti-EBNA-1 antibody titers, while many other estimates are based on measurements of IgG antibodies against EBV VCA. Typically, anti-VCA antibody titers will give a slightly higher estimate, as some anti-VCA positive individuals will subsequently fail to also make anti-EBNA-1 antibodies [25]. In addition, there may be other variations between assays and their cutoff values. When we include the indeterminate samples as seropositive in our analysis, the estimate increases to 70% EBV seropositivity, which is close to estimates for the U.S. general adult population of 73% to 90% [26]. In the replication study (SAFDGS), the same assay yielded a higher seroprevalence estimate (85%). The reason for this difference is unclear, but may be related to simple threshold effects that magnify differences between assays being run at two separate points in time when dichotomizing quantitative assay read-outs. A comparison of our heritability estimates for anti-EBNA-1 antibody titers (h^2^ = 0.43 for the SAFHS, and h^2^ = 0.37 for the SAFDGS) with estimates for anti-VCA antibody titers (h^2^ = 0.32–0.48 [27]) shows that they fall within the same range.

Our study identified multiple, significant associations of anti- EBNA-1 antibody measures with genetic factors located in the HLA region, which contains genes related to immune function in humans. These associations were not found for seroreactivity to 12 other pathogens examined in this study, and therefore appear to be specific to EBNA-1. HLA class I genes are involved in the presentation of peptides (including viral antigens) from within the cell, which attract CD8+ T lymphocytes (cytotoxic T cells) to destroy cells presenting foreign antigens. Previous research identified genetic loci within this region that were associated with the development of infectious mononucleosis upon primary infection with EBV, suggesting that HLA class I polymorphisms influence T cell response during primary EBV infection and viral persistence [17]. The HLA class I region has also been implicated in the development of classical Hodgkin lymphoma among EBV-positive individuals [14]–[16]. HLA class II genes are involved in presenting peptides from outside the cell to CD4+ T lymphocytes (helper T cells), which in turn stimulate B cells to produce antibodies. In our study, genes significantly associated with anti-EBNA-1 antibody levels belong to HLA class II. This supports earlier reports of an association between HLA class II and EBV susceptibility among individuals with multiple sclerosis [17]–[19].

EBV primarily targets resting B cell lymphocytes, which are induced to proliferate, and also infects epithelial cells of the nasopharynx and oropharynx. EBV infects B lymphocytes via attachment to the target cell by binding of the viral major envelope glycoprotein gp350 to complement receptor type two, CD21, on the cell surface [28]. Subsequent penetration of the cell membrane requires a complex of three glycoproteins: gH and gL, which have functional homologs in other herpesviruses; and gp42, which is EBV-specific. Glycoprotein gp42 binds to HLA-DR on the host cell, and in this way HLA class II molecules serve as cofactors for EBV infection of B cells [29]. In our study, we demonstrate a significant association between EBV serostatus and SNP rs477515/rs2516049, which is located in gene *HLA-DRB1* within the HLA-DR gene cluster, and the expression level of *HLA-DRB1* is also significantly correlated with both EBNA-1 serological traits, though we did not observe evidence indicating that the particular EBNA-1-associated SNPs are likely *cis*-acting regulators of this gene. Nearby genes *HLA-DRA* and *HLA-DRB9* do appear to be associated with significant EBNA-1 SNPs, but their expression levels are not significantly correlated with the examined antibody traits. Nonetheless, based on our results, these genes appear to be the best candidates for playing a role in EBV susceptibility in the study population. This may be related to viral penetration of B cells, but it is more likely due to specific haplotype and T cell recognition, as HLA class II genes are also involved in the presentation of viral antigens to T cells, which is important in suppressing proliferation of EBV-infected B cells. HLA-DR is a class II cell surface receptor that serves as a ligand for the T cell receptor. The primary function of HLA-DR is the presentation of peptide antigens to the immune system, and it is closely linked to HLA-DQ, another molecule that presents antigens to T cells. In our study, after conditioning on the top SNP (rs477515/rs2516049) there was significant association of EBNA-1 serostatus and a second independent SNP (rs2854275) that is located within the *HLA-DQB1* gene. After binding to the foreign antigen, T cells stimulate B-cells to produce anti-EBV antibodies. Our finding of significant SNPs located in the *HLA-DR* and *HLA-DQ* genes may relate to the efficiency of cell surface antigen presentation to T cell receptors in EBNA-1 seropositive individuals.

Under normal circumstances, the host immune system is capable of limiting the proliferation of EBV-infected B cells through natural killer (NK) cell and T cell responses. However, some copies of the virus will become latent in memory B cells, at concentrations of approximately 1 to 50 per 10^6^ in cells within peripheral blood in healthy individuals [30]. Although most individuals will not experience clinical symptoms after EBV enters latency, a small percentage may develop cancer following a re-activation of infection. During active infection, the virus produces approximately 100 different viral proteins that are involved in viral replication and modulating the immune response in the host. However, during latent infection only about 10 proteins are produced by the virus, including EBNA-1. This protein, which was used in our study to quantify EBV antibody titer, correlates closely with past infection [31] and is expressed on all EBV-associated tumors. EBNA-1 is suggested to elicit poor CD8+ T cell response [32] and it is considered to be a universal viral oncogene [2]. EBV-associated malignancies are higher in particular geographic locations as well as among certain ethnic groups, indicating that both environmental exposures and genetic factors are likely involved in disease risk. Cancers linked to EBV infection include: Burkitt lymphoma, which is prevalent in Africa and for which malaria may be a cofactor [6]; nasopharyngeal carcinoma, which is more common among individuals from South China [33], [34]; Hodgkin lymphoma, which is reported to have a higher incidence in Hispanic and Asian/Pacific Islander populations [35]; parotid tumors in patients from Alaska [8]; and some gastric cancers [9]. EBV infection is also associated with post-transplant lymphoproliferative disorders [36]. Serological evidence points to high EBNA-1 antibody titers prior to the onset of clinical symptoms for several of these malignancies [7], [37]. In our study, we found an overlap between EBNA-1 traits and NPC susceptibility loci in *HLA-A* and *HLA-DR/DQ* genes, and with HL loci in *BTNL2*, and *HLA-DR* genes. It has been suggested that the presentation of EBV-derived peptides is in some way involved in the pathogenesis of EBV-related cancer [16]. We also found suggestive evidence for association of top SNPs with the expression of *EGFL8*, which has previously been implicated in cancer progression, and *NCR3*, a gene that encodes a natural cytotoxicity receptor that may aid NK cells in lysing tumor cells.

Studies indicate that EBV may be implicated in the development of autoimmune disease, including systemic lupus erythematosus (SLE) and multiple sclerosis (MS) [38], [39]. In SLE, patients may be unable to keep latent EBV infection in check, possibly due to defective T cell response to the virus. Molecular mimicry appears to play a role in SLE autoimmunity, as humoral immune response initially targets the proline-rich repeat motif PPPGMRPP, antibodies against which also cross-react with the EBNA-1 peptide PPPGRRP [40], [41]. In our study we provide evidence for shared genetic factors that influence both anti-EBNA-1 antibody status and autoimmunity, as shown by a significant association between EBNA-1 serological measures and SLE SNPs rs3135391, rs9268832 and rs9271366, all located in the HLA region. SNP rs9268832 was both the top SNP associated with the EBNA-1 discrete serostatus trait in our study of Mexican Americans and the top SNP identified in a Spanish SLE cohort [42]. Given that this SNP lies within the HLA class II pseudogene *DRB9*, it has been suggested that this association is due to the composite effect of SNPs rs3130490 (located in the *MSH5* gene) and rs3129768 (located between *HLA-DRB1* and *HLA-DQA1* genes). Dysregulation of the *MSH5* gene, which plays a role in immunoglobulin class switching, allowing B cells to generate different classes of antibody but with the same specificity, is proposed to contribute to risk of developing SLE. While SNPs located within this gene were not statistically significant in our sample after adjusting for multiple testing, other genetic factors that appear to influence both EBNA-1 serostatus and SLE include *HLA-DR* and *HLA-DQ* loci, possibly due to mechanisms shared across various autoimmune and inflammatory diseases.

Susceptibility to MS was previously shown to be associated with HLA genes, and with *HLA-DRB1* in particular [43], which in our study was significantly related to EBNA-1 serostatus. Indeed, our results, which are based on genome-wide investigation, support an earlier report of association between anti-EBNA-1 antibody titer level and *HLA-DRB1* in an MS cohort [18]. That study observed that HLA-DRB1*15 positive individuals had a higher level of anti-EBNA-1 antibody titer and greater risk of developing MS, indicating that HLA genetic influence on MS risk may also involve control of EBV infection. In addition to *HLA-DRB1*, MS has been associated with changes in the expression of a number of other genes, including other *HLA-DR* genes and *HLA-DQ* genes [44]. We found evidence for a significant overlap between anti-EBNA-1 antibodies and three MS HLA SNPs (rs3129860, rs3135388, and rs9271366), which are associated with *HLA-DR* and *HLA-DQ* genes, and may be related to a more general autoimmune/inflammatory response.

In summary, the results of our study indicate that genetic determinants in the HLA region are important in the immune response to EBV and subsequent regulation of infection with this pathogen. Variation in EBNA-1 antibody titer among individuals may be due in part to variation in B cell permeability to EBV infection and/or differences in cell surface antigen presentation, as indicated by a statistically significant relationship between genetic factors within the HLA II region and EBV serostatus (defined here by the level of anti-EBNA-1 antibodies) that was not found for the other pathogens examined. Further investigation may reveal the underlying mechanisms by which these HLA genes potentially limit EBV viral load, possibly influencing risk for developing autoimmune disease or cancer in infected individuals.

## Methods

### Ethics statement

The study and protocols were approved by the Institutional Review Board at the University of Texas Health Science Center at San Antonio, and informed consent was obtained from all participants.

### Study population

Individuals in this study included 1,367 members of extended, multi-generational families from the Mexican American community in San Antonio, Texas, and surrounding region. They were recruited during the years 1991–1995 for participation in the San Antonio Family Heart Study (SAFHS), which seeks to identify genetic risk factors for cardiovascular disease [45], and were ascertained without regard to any specific disease phenotype. Participants included 551 men and 816 women, who ranged in age from 16–94 years and represented 63 families (Table S1). These families had up to 6 generations and the largest consisted of 101 phenotyped individuals. Included in the study were 267 sibships, with an average size of 3.3 and size range of 2–12.

Significant findings were replicated for a separate sample of 589 Mexican Americans participating in the San Antonio Diabetes/Gallbladder Study (SAFDGS). The SAFDGS seeks to investigate the genetic influences underlying type II diabetes mellitus and gallbladder disease, and it is similar in design to the SAFHS except that recruitment was based on a single diabetic proband in each pedigree, and the sample is therefore enriched for diabetics [21], [46]. Please note that this is a very weak form of ascertainment in Mexican Americans from San Antonio, where lifetime prevalence of diabetes approaches 30%. In fact, 20 years after the initiation of both studies, the prevalence rates of major diseases, such as heart disease, diabetes, and obesity, are not significantly different between these two component studies. The SAFDGS participants consisted of 39 families, representing up to 6 generations, and included 115 sibships, which ranged in size from 2–9 (average size of 3.2). Analyses were also run on the combined data set (SAFHS+SAFDGS), which included 1,956 phenotyped individuals. Familial relationships were confirmed based on genotype composition using PREST [47].

### Sample collection and determination of serostatus

Blood samples were collected from participants after an overnight fast, at the time of recruitment (1991–1995) using EDTA vacutainers. Frozen plasma aliquots were obtained as previously described [48], along with the buffy coat for DNA extraction, and stored at −80°C. Plasma samples were thawed just prior to antibody determinations, and IgG antibodies to Epstein-Barr virus nuclear antigen 1 (EBNA-1) were measured using a commercially available enzyme-linked immunosorbent assay (ELISA) kit (IBL-America, Minneapolis, MN). Seropositive/seronegative status was determined according to the manufacturer's instructions using the following absorbance values: seronegative if ≤0.9; indeterminate if >0.9 and <1.1; and seropositive if ≥1.1. Antibody titers to 12 comparative pathogens were also obtained and included: *Chlamydophila* pneumoniae, Helicobacter pylori, Toxoplasma gondii, cytomegalovirus (CMV), herpes simplex type I virus (HSV-1), herpes simplex type II virus (HSV-2), human herpesvirus 6 (HHV-6), varicella zoster virus (VZV), adenovirus 36 (Ad36), hepatitis A virus (HAV), influenza A virus, and influenza B virus [23]. For Ad36, a previously described serum neutralization test was utilized for measuring antibodies [49], and analyses were run in duplicate, with specimens assigned as seropositive if both replicates had neutralization titers ≥1∶8, otherwise they were considered to be seronegative. Serostatus for all other pathogens was determined using the same criteria as for EBNA-1 (i.e., seronegative if ≤0.9; indeterminate if >0.9 and <1.1; and seropositive if ≥1.1 [23]).

### SNP genotyping

DNA was extracted from the lymphocyte samples from study participants. SNPs were typed using several versions of Illumina's SNP genotyping BeadChip microarrays (HumanHap550v3, HumanExon510Sv1, Human1Mv1 and Human1M-Duov3), according to the Illumina Infinium protocol (Illumina, San Diego, CA). SNP genotype data underwent extensive processing prior to analysis: SNPs with a low call rate, that were monomorphic or those comprising <10 individuals with the minor allele were excluded from analysis. Additional SNPs were excluded if Hardy-Weinberg Equilibrium test statistics were equivalent to p≤10^−4^ (calculated using SOLAR [50] while taking relationships properly into account), leaving a total of 944,565 SNPs for further analysis. Allele frequencies were computed using maximum likelihood estimates in SOLAR [50]. SNP genotypes were checked for Mendelian consistency using Simwalk [51]. MERLIN [52] was used to impute missing genotypes conditional on relatives' genotypes, with a weighted average of possible genotypes being used when an individual's genotype could not be inferred with certainty. Multipoint identity-by-descent (MIBD) matrices, based on a subset of 28,219 informative SNPs that were not in LD with one another, were calculated with LOKI [53]. The chromosomal maps used in the analyses were based on those generated by deCODE genetics [54].

### Statistical analysis

Both anti-EBNA-1 quantitative antibody titer and discrete serostatus traits were analyzed. Statistical analyses of the sample of related individuals were performed using a variance components (VC) approach with the computer software package SOLAR [50]. Due to the sensitivity of VC analyses to non-normality, the quantitative antibody titer trait was transformed prior to analysis using an inverse, rank-based normalization to ensure a standard normal distribution of this phenotype. For the genetic analysis of the discrete EBNA-1 serostatus trait within a VC framework, a liability threshold model was used, in which serostatus was assumed to reflect an unobservable underlying quantitative liability, with individuals above a threshold being seropositive, and those below being seronegative [55], [56], individuals with indeterminate serostatus were excluded from analysis. All analyses included sex, age (at the time of sample collection), and their interactions as covariates. Although the SAFDGS was enriched for diabetics, diabetes status was found to not be a significant predictor of EBNA-1 antibody status and therefore was not included in further analyses. Narrow-sense heritability, or the proportion of phenotypic variance attributable to the aggregate effects of additive genetic variation, was estimated along with the influence of shared environmental factors, which were modeled using a “household” random effects component [20]. Individuals living together at the time of the blood draw were considered members of the same household. Details concerning the length of cohabitation, however, were not available. Because the SAFHS includes extended families, which provide information on both linkage and association, we performed several analyses in order to maximize the amount of information obtained from this sample. We performed genome-wide linkage analysis, using MIBDs based on 28,219 SNPs, to identify regions of the genome that may harbor genetic variants influencing EBNA-1 serological traits. We also performed joint genome-wide linkage and association analysis, based on 944,565 SNPs that were available for 1,367 individuals, in order to have more power for localizing the responsible loci. The joint analysis was conducted under a VC model in which the linkage component was implemented as a regular VC-based random effects linkage model, and an additive measured genotype model was used for the association component. Two-times the natural logarithm of the likelihood ratio of the joint linkage and association test was assumed to be distributed as a 50∶50 mixture of chi-squared random variables with 1 and 2 degrees of freedom, respectively. In order to remove the long-distance linkage effect and hone in on the shorter-range association signal, and thus achieve better differentiation among SNPs, we then performed association conditional on linkage for the extended HLA region (nucleotide positions 29,677,984 to 33,485,635), based on all 5689 available SNPs. As population substructure may result in spurious associations in GWAS studies, we corrected for this by using principal components analysis to model differences in ancestral contributions among study participants [57]. R princomp [58] was used to run the principal components analysis on a subset of 11,512 autosomal SNPs (determined to be in low mutual linkage distribution [LD]) in 345 genotyped founders, and offspring were assigned PC values averaged over their parents, in order to not accidentally remove true pedigree differences. The first five principal components were included as additional covariates in all statistical analyses (these account for ∼3% of the variance in the genotype scores, indicating that there is in fact little evidence for stratification in the Mexican American cohort). Given that there is a large amount LD in the HLA region on chromosome 6, we ran multiple conditional analyses on the top EBNA-1 SNPs, in order to determine the number of independent significantly-associated SNPs. In addition, LD specific to the Mexican American study population, and appropriately taking the relatedness into account, was calculated using SOLAR [50] and regional plots, based on this information, were generated using LocusZoom [59].

### Transcriptional profiles

Transcriptional profile data were available for PBMCs from 1,243 study participants, collected at the same time as the plasma samples used for EBNA-1 screening, as previously described [60]. Raw and normalized expression values are available under the accession number E-TABM-305 at: http://www.ebi.ac.uk/arrayexpress. Briefly, sample quality was examined by comparing the number of expressed probes (p≤0.05), mean expression across expressed probes, and mean correlation (across expressed probes) with other samples, and 1,243 samples were deemed to give high quality expression profiles. Transcripts with significant expression at a false discovery rate (FDR) ≤0.05 were identified using a one-sided binomial test (based on counts of samples with successful and unsuccessful detection at p≤0.05), yielding 20,634 significantly detected probes. Subsequently, we performed “background noise correction”, log2 transformation, and quantile normalization.

We then tested whether SNPs that were significantly associated with EBNA-1 antibody measurements were also significantly associated with the quantitative expression levels of neighboring transcripts (i.e., whether the candidate SNPs are putative *cis*-regulatory variants), and whether those transcripts were in turn associated with EBNA-1 antibody measures. Prior to these analyses, in order to detect and remove the impact of suspected as well as unknown confounding variables on expression levels, we performed principal components (PC) analysis (after inverse, rank-based normalization of transcripts) on the expression profile data (details on methodology are being prepared for publication elsewhere). For detection of putative *cis*-acting expression quantitative trait nucleotides, the top 50 expression PCs were regressed out, followed by additive measured-genotype-based association analysis (conditional on linkage) on transcripts in the HLA region. Before correlating expression levels with anti-EBNA-1 traits, we examined (by regression analysis) the relationship between each of the top 50 expression PCs to the antibody traits, and regressed out all of the top 50 PCs except those that were significantly related to the antibody traits (so that we would not accidentally remove any true connection between expression and antibody traits).

## Supporting Information

Figure S1 Linkage results for EBNA-1 quantitative (blue) and discrete (red) serostatus traits for SAFHS. (A) Genome-wide linkage. (B) Chromosome 6 linkage.
(TIF)

Figure S2 Quantile–quantile plot of genome-wide association results (conditional on linkage). (A) Including the extended HLA region (genomic inflation factor^a^ λ = 1.02). ^a^Devlin, B. & Roeder, K. Genomic control for association studies. *Biometrics* 55, 997–1004 (1999). (B) Excluding the extended HLA region (λ = 1.00).
(TIF)

Figure S3 Linkage results for EBNA-1 quantitative (blue) and discrete (red) serostatus traits for SAFDGS. (A) Genome-wide linkage. (B) Chromosome 6 linkage.
(TIF)

Figure S4 Genome-wide joint linkage and association analysis results for EBNA-1 antibody traits for SAFDGS. (A) Quantitative antibody titer. (B) Discrete serostatus.
(TIF)

Figure S5 Association analysis results, given linkage, conditional on SNPs rs477515/rs2516049. Results are for extended HLA region in the combined sample (SAFHS+SAFDGS). The LD pattern was estimated based on SNP genotypes from study participants. SNPs in red are highly correlated with the top SNP associated with the EBNA-1 quantitative trait (rs2854275). (A) Quantitative antibody trait. (B) Discrete serostatus trait.
(TIF)

Figure S6 Pattern of linkage disequilibrium. Shown are results for the 41 SNPs significantly associated with the EBNA-1 serological traits in the SAFHS+SAFDGS (presented in Table 2 and Table 3). Red indicates highly correlated SNPs.
(TIF)

Table S1 Information on pedigree relationships. Included are participants in the San Antonio Family Heart Study (SAFHS) and San Antonio Family Diabetes/Gallbladder Study (SAFDGS).
(DOCX)

Table S2 Genome-wide joint linkage and association analysis. Shown are all SNPs yielding genome-wide significant *p*-values with either the quantitative and/or the qualitative antibody phenotype in the SAFHS. The regression coefficients refer to the estimated change in the phenotype for each dose of the rarer SNP allele. For the SAFHS all genome-wide significant results (*p*≤5.29×10^−8^) are presented in bold lettering. After correcting for multiple testing during replication in the SAFDGS (we tested the entire HLA region, with 5689 available SNPs: *p*≤0.05/5689≈8.79×10^−6^), 10 SNPs are significant for the replicate sample. When using the combined sample of both studies (SAFHS+SAFDGS), all SNPs originally significant in the SAFHS discovery sample are highly significant.
(DOCX)

Table S3 Association, conditional on linkage, results for SNP-transcript pairs in the HLA region. Focus is on the 41 SNPs previously found to be significantly associated with EBNA-1 traits (presented in Table 2 and Table 3). Shown are results for the top 74 pairs (*p*≤1.0×10^−2^). Only four SNP-transcript pairs were significant after adjusting for multiple testing.
(DOCX)

Table S4 Genetic correlations between HLA transcripts and EBNA-1 serological traits. Only one transcript was significant, after adjusting for multiple testing.
(DOCX)
