# Supplementary figures and images for: A Genome-Wide Integrative Genomic Study Localizes Genetic Factors Influencing Antibodies against Epstein-Barr Virus Nuclear Antigen 1 (EBNA-1)
Source: PLoS Genet. 2013 Jan 10;9(1):e1003147. doi: 10.1371/journal.pgen.1003147 (PMC3542101; doi:10.1371/journal.pgen.1003147)

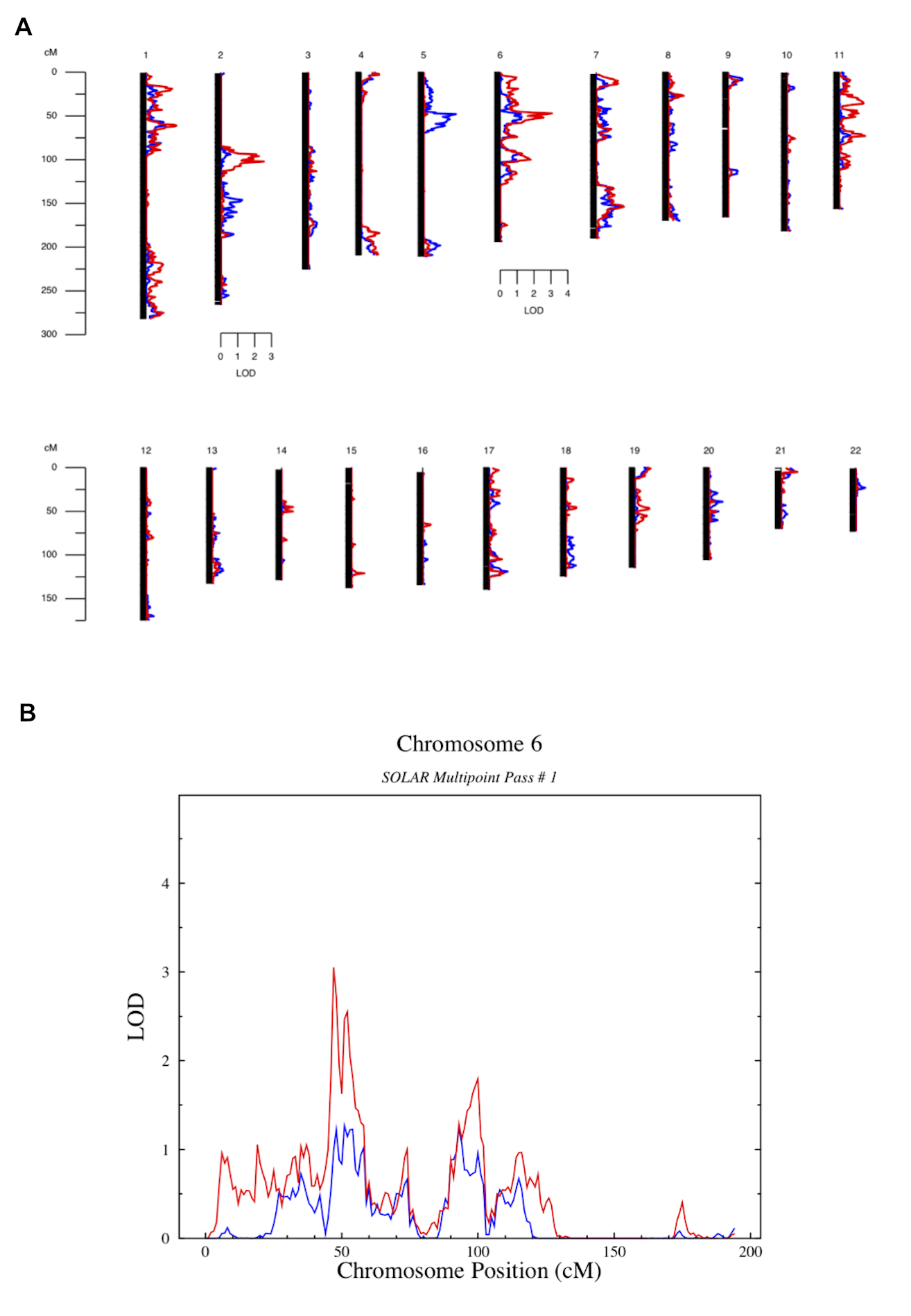

Supplement: Figure S1 — Linkage results for EBNA-1 quantitative (blue) and discrete (red) serostatus traits for SAFHS. (A) Genome-wide linkage. (B) Chromosome 6 linkage. (TIF) [file pgen.1003147.s001.tif]

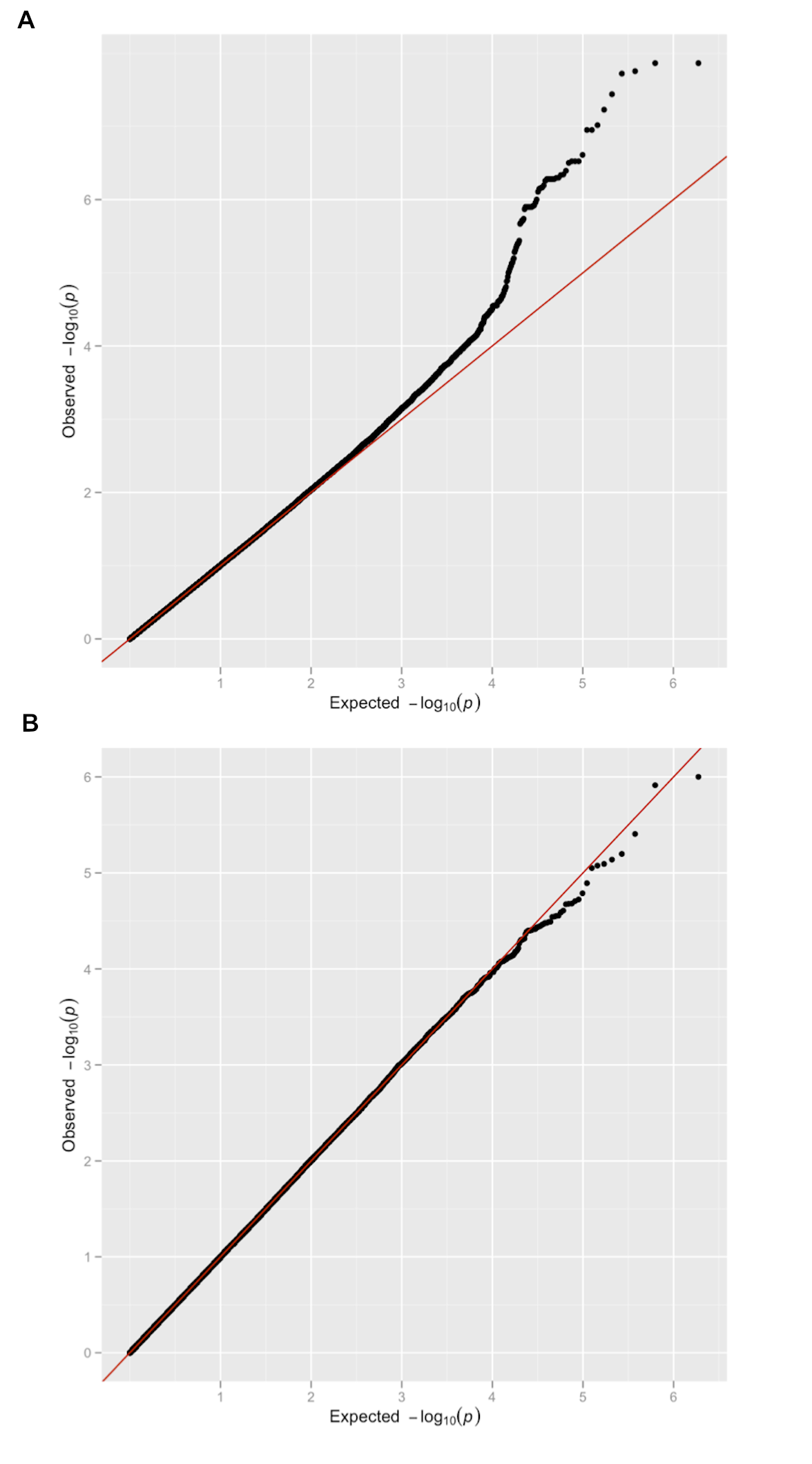

Supplement: Figure S2 — Quantile–quantile plot of genome-wide association results (conditional on linkage). (A) Including the extended HLA region (genomic inflation factora λ = 1.02). aDevlin, B. & Roeder, K. Genomic control for association studies. Biometrics 55, 997–1004 (1999). (B) Excluding the extended HLA region (λ = 1.00). (TIF) [file pgen.1003147.s002.tif]

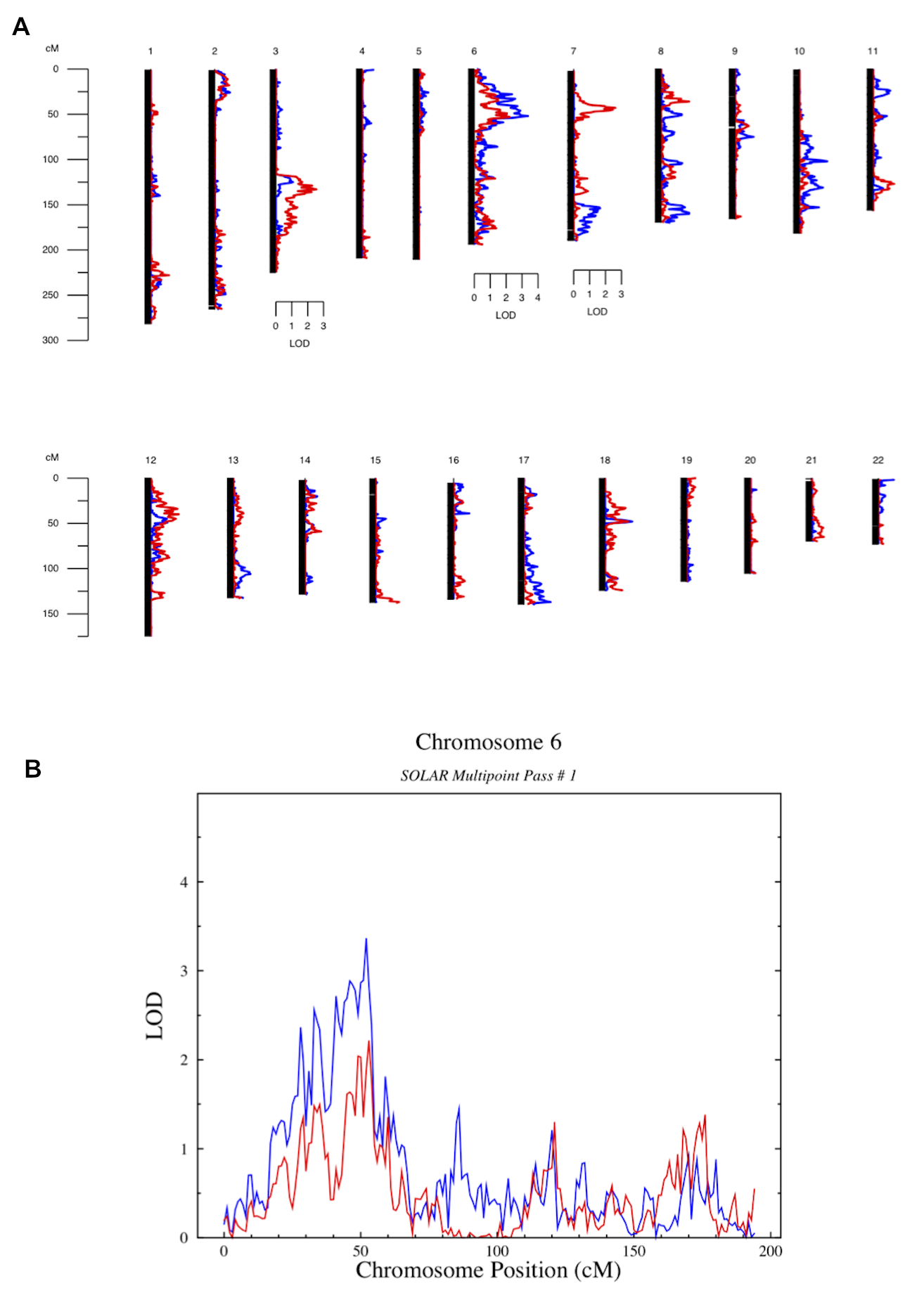

Supplement: Figure S3 — Linkage results for EBNA-1 quantitative (blue) and discrete (red) serostatus traits for SAFDGS. (A) Genome-wide linkage. (B) Chromosome 6 linkage. (TIF) [file pgen.1003147.s003.tif]

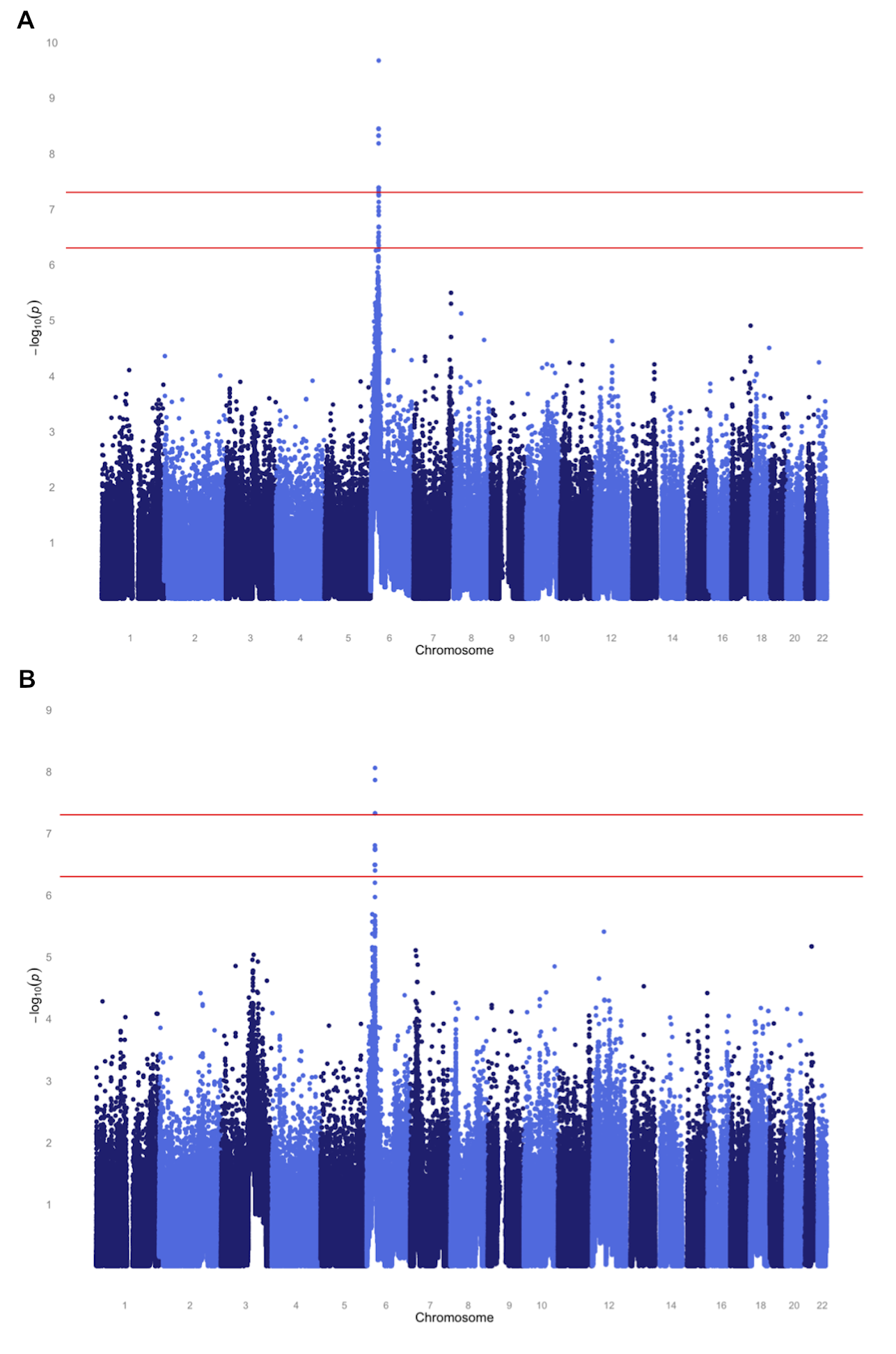

Supplement: Figure S4 — Genome-wide joint linkage and association analysis results for EBNA-1 antibody traits for SAFDGS. (A) Quantitative antibody titer. (B) Discrete serostatus. (TIF) [file pgen.1003147.s004.tif]

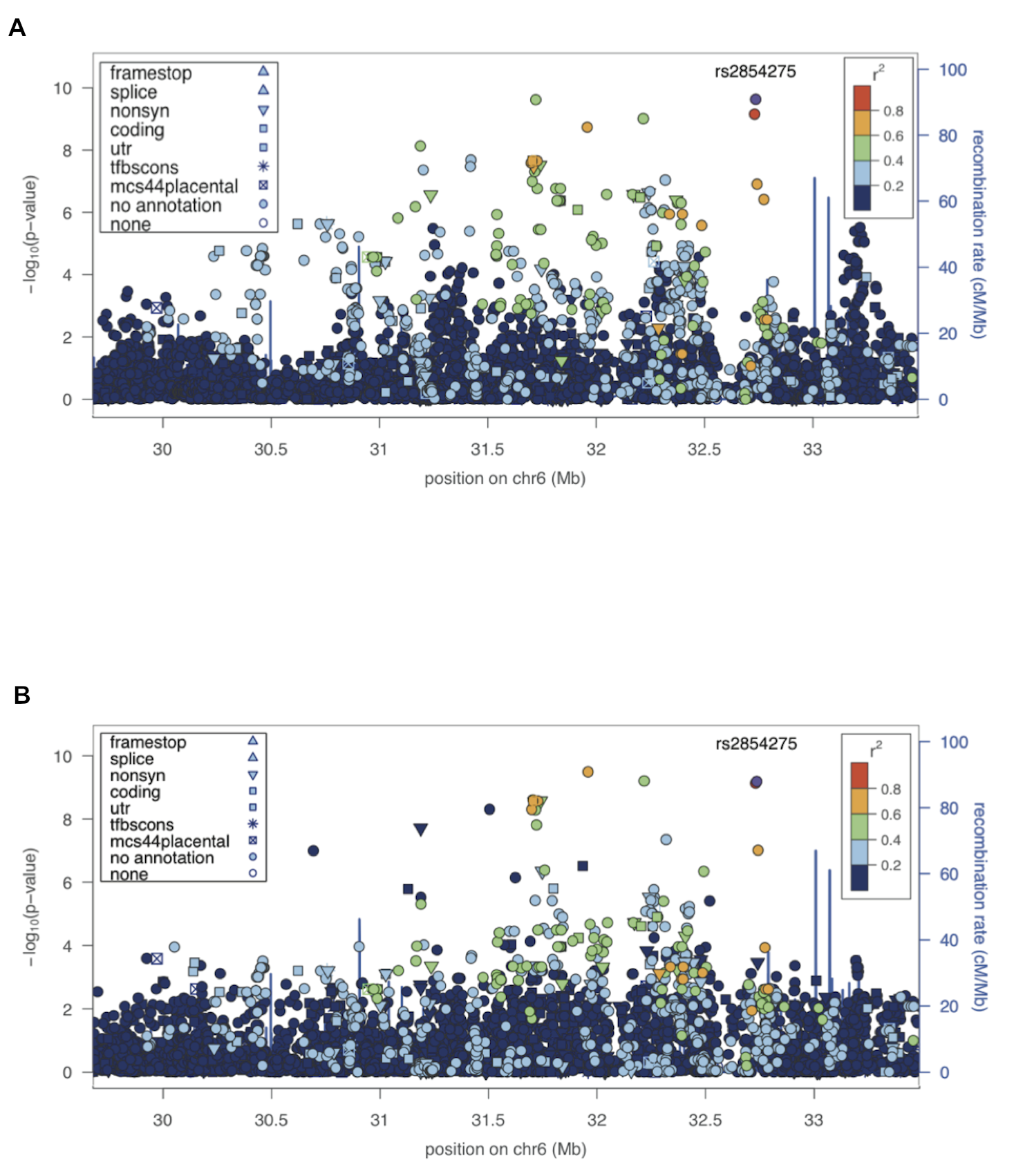

Supplement: Figure S5 — Association analysis results, given linkage, conditional on SNPs rs477515/rs2516049. Results are for extended HLA region in the combined sample (SAFHS+SAFDGS). The LD pattern was estimated based on SNP genotypes from study participants. SNPs in red are highly correlated with the top SNP associated with the EBNA-1 quantitative trait (rs2854275). (A) Quantitative antibody trait. (B) Discrete serostatus trait. (TIF) [file pgen.1003147.s005.tif]

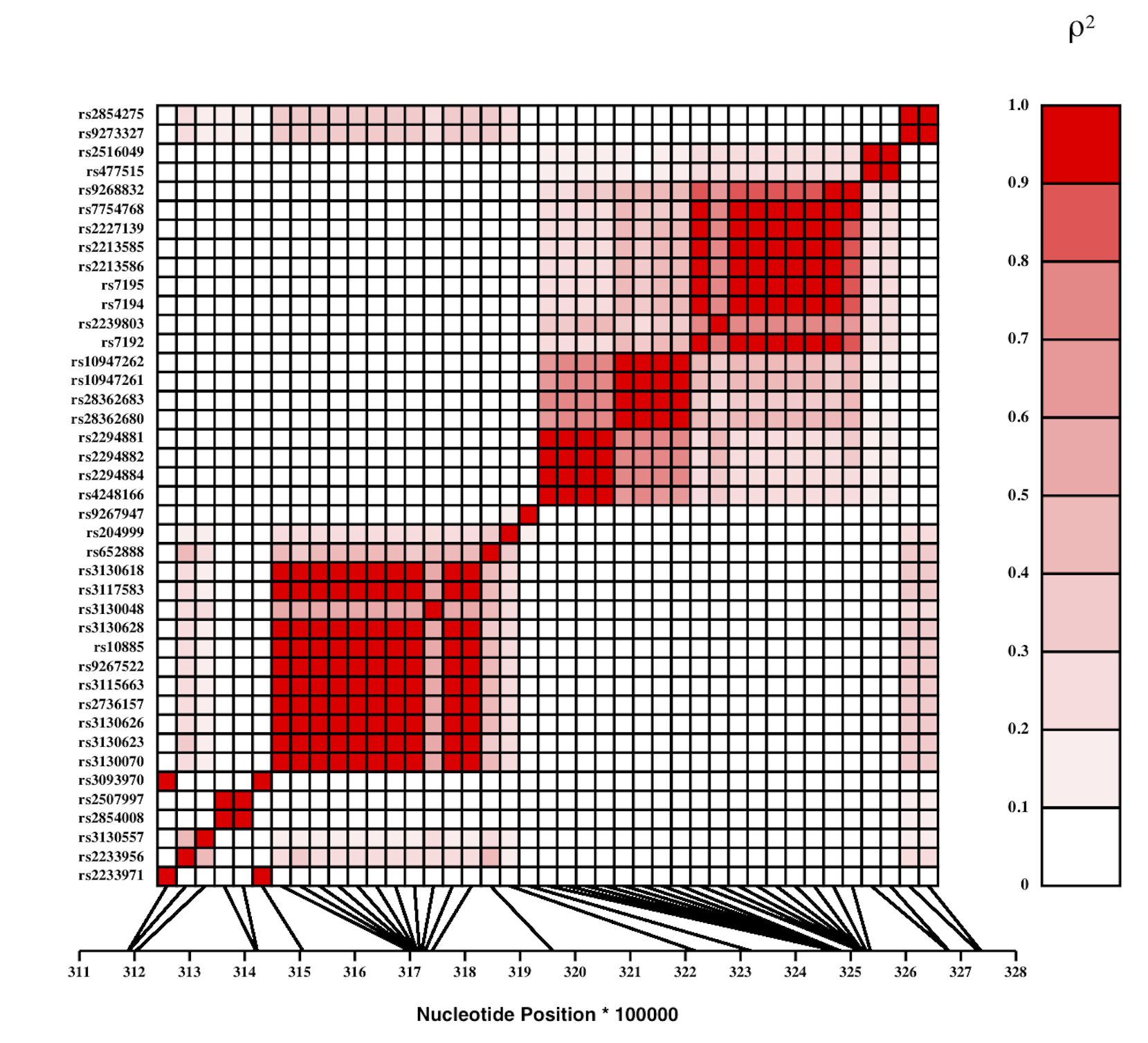

Supplement: Figure S6 — Pattern of linkage disequilibrium. Shown are results for the 41 SNPs significantly associated with the EBNA-1 serological traits in the SAFHS+SAFDGS (presented in Table 2 and Table 3). Red indicates highly correlated SNPs. (TIF) [file pgen.1003147.s006.tif]
