# Supplementary material for: A Genome-Wide Integrative Genomic Study Localizes Genetic Factors Influencing Antibodies against Epstein-Barr Virus Nuclear Antigen 1 (EBNA-1)
Source: PLoS Genet. 2013 Jan 10;9(1):e1003147. doi: 10.1371/journal.pgen.1003147 (PMC3542101; doi:10.1371/journal.pgen.1003147)
Supplement: Table S1 — Information on pedigree relationships. Included are participants in the San Antonio Family Heart Study (SAFHS) and San Antonio Family Diabetes/Gallbladder Study (SAFDGS). (DOCX) [file pgen.1003147.s007.docx]

**Table S1. Information on pedigree relationships.** Included are participants in the San Antonio Family Heart Study (SAFHS) and San Antonio Family Diabetes/Gallbladder Study (SAFDGS).

|  | SAFHS | SAFDGS |
| --- | --- | --- |
| Pedigree information |  |  |
| Number of pedigrees | 63 | 39 |
| Maximum number of generations | 6 | 6 |
| Size of largest pedigree, n | 101 | 41 |
| Number of sibships | 267 | 115 |
| Average sibship size (range) | 3.3 (2-12) | 3.2 (2-9) |
| Familial relationships, observed pairs |  |  |
| Parent-offspring | 1,211 | 409 |
| Monozygotic twins | 3 | 1 |
| Full siblings | 1,434 | 528 |
| Half siblings | 201 | 89 |
| Grandparent-grandchild | 451 | 108 |
| Avuncular | 2,785 | 765 |
| Half avuncular | 411 | 95 |
| First cousins | 3,067 | 752 |

Table values represent phenotyped individuals only. Actual pedigree sizes and pairs of familial relationships may be larger due to the presence of unphenotyped individuals
