# Supplementary material for: A Genome-Wide Integrative Genomic Study Localizes Genetic Factors Influencing Antibodies against Epstein-Barr Virus Nuclear Antigen 1 (EBNA-1)
Source: PLoS Genet. 2013 Jan 10;9(1):e1003147. doi: 10.1371/journal.pgen.1003147 (PMC3542101; doi:10.1371/journal.pgen.1003147)
Supplement: Table S2 — Genome-wide joint linkage and association analysis. Shown are all SNPs yielding genome-wide significant p-values with either the quantitative and/or the qualitative antibody phenotype in the SAFHS. The regression coefficients refer to the estimated change in the phenotype for each dose of the rarer SNP allele. For the SAFHS all genome-wide significant results (p≤5.29×10^−8^) are presented in bold lettering. After correcting for multiple testing during replication in the SAFDGS (we tested the entire HLA region, with 5689 available SNPs: p≤0.05/5689≈8.79×10^−6^), 10 SNPs are significant for the replicate sample. When using the combined sample of both studies (SAFHS+SAFDGS), all SNPs originally significant in the SAFHS discovery sample are highly significant. (DOCX) [file pgen.1003147.s008.docx]

**Table S2.** **Genome-wide joint linkage and association analysis**. Shown are all SNPs yielding genome-wide significant *p*-values with either the quantitative and/or the qualitative antibody phenotype in the SAFHS. The regression coefficients refer to the estimated change in the phenotype for each dose of the rarer SNP allele. For the SAFHS all genome-wide significant results (*p* ≤ 5.29x10^-8^) are presented in bold lettering. After correcting for multiple testing during replication in the SAFDGS (we tested the entire HLA region, with 5689 available SNPs: *p* ≤ 0.05/5689 ≈ 8.79x10^-6^), 10 SNPs are significant for the replicate sample. When using the combined sample of both studies (SAFHS + SAFDGS), all SNPs originally significant in the SAFHS discovery sample are highly significant.

| SNP | Location on chrom 6 (bp) | Nearest gene | This study (SAFHS) | | Replicate (SAFDGS) | | Combined (SAFHS + SAFDGS) | |
| --- | --- | --- | --- | --- | --- | --- | --- | --- |
|  |  |  | Quantitative  *p*-value (ß_SNP_) | Discrete  *p*-value (ß_SNP_)^a^ | Quantitative  *p*-value (ß_SNP_) | Discrete  *p*-value (ß_SNP_)^a^ | Quantitative  *p*-value (ß_SNP_) | Discrete  *p*-value (ß_SNP_)^a^ |
| rs3132451 | 31690004 | *AIF1* | 7.13x10^-8^ (-0.38) | **4.04x10^-9^ (0.59)** | 6.70x10^-4^ (0.05) | 2.62x10^-3^ (0.06) | **2.78x10^-6^ (-0.15)** | **4.75x10^-6^ (0.16)** |
| rs3130070 | 31699787 | *BAT2* | 2.71x10^-7^ (-0.37) | **6.87x10^-9^(0.59)** | 2.53x10^-4^ (-0.18) | 3.55x10^-4^ (0.36) | **1.39x10^-9^ (-0.32)** | **6.78x10^-11^ (0.48)** |
| rs3130622 | 31700503 | *BAT2* | 1.56x10^-7^ (-0.38) | **6.39x10^-9^ (0.60)** | 6.46x10^-4^ (0.05) | 2.66x10^-3^ (0.06) | **4.94x10^-6^ (-0.14)** | **7.08x10^-6^ (0.14)** |
| rs3130623 | 31705679 | *BAT2* | 3.36x10^-6^ (-0.32) | **1.74x10^-8^ (0.56)** | 1.31x10^-4^ (-0.22) | 1.56x10^-4^ (0.41) | **5.07x10^-9^ (-0.29)** | **4.37x10^-11^ (0.48)** |
| rs3130626 | 31706468 | *BAT2* | 2.71x10^-7^ (-0.37) | **6.87x10^-9^ (0.59)** | 2.18x10^-4^ (-0.19) | 2.56x10^-4^ (0.38) | **1.07x10^-9^ (-0.32)** | **3.51x10^-11^ (0.49)** |
| rs2736157 | 31708799 | *BAT2* | 2.71x10^-7^ (-0.37) | **6.87x10^-9^ (0.59)** | 2.18x10^-4^ (-0.19) | 2.56x10^-4^ (0.38) | **1.07x10^-9^ (-0.32)** | **3.51x10^-11^ (0.49)** |
| rs3115663 | 31709822 | *BAT2* | 2.71x10^-7^ (-0.37) | **6.87x10^-9^ (0.59)** | 2.16x10^-4^ (-0.20) | 2.52x10^-4^ (0.39) | **1.06x10^-9^ (-0.32)** | **3.43x10^-11^ (0.49)** |
| rs9267522 | 31711749 | *BAT2* | 2.71x10^-7^ (-0.37) | **6.87x10^-9^ (0.59)** | 2.18x10^-4^ (-0.19) | 2.56x10^-4^ (0.38) | **1.07x10^-9^ (-0.32)** | **3.51x10^-11^ (0.49)** |
| rs10885 | 31712570 | *BAT2* | 2.71x10^-7^ (-0.37) | **6.87x10^-9^ (0.59)** | 2.62x10^-4^ (-0.18) | 2.36x10^-4^ (0.38) | **1.57x10^-9^ (-0.31)** | **3.78x10^-11^ (0.49)** |
| rs3130628 | 31717251 | *BAT3* | 4.55x10^-7^ (-0.36) | **8.24x10^-9^ (0.58)** | 2.73x10^-4^ (-0.18) | 4.01x10^-4^ (0.34) | **2.61x10^-9^ (-0.31)** | **7.25x10^-11^ (0.48)** |
| rs3130048 | 31721718 | *BAT3* | **4.62x10^-9^ (-0.35)** | **2.18x10^-9^ (0.51)** | 3.14x10^-5^ (-0.24) | 4.54x10^-4^ (0.28) | **3.77x10^-12^ (-0.31)** | **3.11x10^-11^ (0.41)** |
| rs3117583 | 31727555 | *BAT3* | 2.71x10^-7^ (-0.37) | **6.87x10^-9^ (0.59)** | 2.18x10^-4^ (-0.19) | 2.56x10^-4^ (0.38) | **1.07x10^-9^ (-0.32)** | **3.51x10^-11^ (0.49)** |
| rs3130618 | 31740113 | *BAT4* | 3.91x10^-7^ (-0.36) | **6.87x10^-9^ (0.59)** | 2.18x10^-4^ (-0.19) | 2.56x10^-4^ (0.38) | **1.51x10^-9^ (-0.32)** | **3.51x10^-11^ (0.49)** |
| rs9267532 | 31747958 | *LY6G5B* | 4.68x10^-6^ (-0.41) | **4.54x10^-8^ (0.68)** | 7.36x10^-4^ (-0.10) | 1.04x10^-3^ (0.34) | **1.40x10^-7^ (-0.32)** | **2.37x10^-10^ (0.58)** |
| rs9267536 | 31759173 | *LY6G5C* | 2.00x10^-6^ (-0.43) | **4.85x10^-8^ (0.69)** | 7.90x10^-4^ (-0.09) | 9.57x10^-4^ (0.39) | **7.65x10^-8^ (-0.35)** | **1.70x10^-10^ (0.62)** |
| rs652888 | 31959213 | *EHMT2* | 1.53x10^-7^ (-0.36) | **1.02x10^-9^ (0.60)** | 1.65x10^-5^ (-0.33) | 1.00x10^-4^ (0.46) | **6.12x10^-11^ (-0.34)** | **8.41x10^-13^ (0.54)** |
| rs204999 | 32217957 | *PRRT1* | **8.68x10^-9^ (-0.30)** | **1.61x10^-10^ (0.48)** | **5.39x10^-8^ (-0.37)** | **1.56x10^-7^ (0.56)** | **6.30x10^-15^ (-0.32)** | **7.10x10^-16^ (0.48)** |
| rs204995 | 32262263 | *PBX2* | 2.60x10^-7^ (-0.30) | **5.44x10^-9^ (0.48)** | 9.81x10^-5^ (-0.18) | 4.69x10^-5^ (0.39) | **4.14x10^-10^ (-0.26)** | **3.41x10^-11^ (0.40)** |
| rs204994 | 32262976 | *PBX2* | 8.76x10^-7^ (-0.30) | **1.30x10^-8^ (0.49)** | 5.53x10^-5^ (-0.22) | 2.29x10^-5^ (0.43) | **6.28x10^-10^ (-0.27)** | **4.33x10^-11^ (0.41)** |
| rs204992 | 32264886 | *PBX2* | 2.60x10^-6^ (-0.28) | **1.85x10^-8^ (0.48)** | 3.54x10^-5^ (-0.23) | 1.50x10^-5^ (0.46) | **9.12x10^-10^ (-0.27)** | **3.23x10^-11^ (0.41)** |
| rs4248166 | 32474399 | *C6orf10* | **4.29x10^-9^ (0.29)** | **2.54x10^-10^(-0.45)** | **6.62x10^-6^ (0.22)** | 6.14x10^-4^ (-0.21) | **2.13x10^-13^ (0.27)** | **3.96x10^-13^ (-0.39)** |
| rs2294884 | 32475237 | *BTNL2* | 5.58x10^-8^ (0.26) | **1.71x10^-9^ (-0.42)** | 1.02x10^-5^ (0.21) | 9.68x10^-4^ (-0.18) | **3.44x10^-12^ (0.25)** | **6.07x10^-12^ (-0.36)** |
| rs2294882 | 32475493 | *BTNL2* | **2.61x10^-8^ (0.26)** | **1.73x10^-10^ (-0.44)** | 1.06x10^-5^ (0.21) | 8.38x10^-4^ (-0.18) | **1.83x10^-12^ (0.25)** | **6.37x10^-13^ (-0.38)** |
| rs2294881 | 32475582 | *BTNL2* | **2.61x10^-8^ (0.26)** | **1.73x10^-10^ (-0.44)** | 1.06x10^-5^ (0.21) | 8.38x10^-4^ (-0.18) | **1.83x10^-12^ (0.25)** | **6.37x10^-13^ (-0.38)** |
| rs28362680 | 32478794 | *BTNL2* | 1.23x10^-7^ (0.26) | **3.12x10^-10^ (-0.47)** | 2.90x10^-5^ (0.19) | 1.26x10^-3^ (-0.16) | **2.97x10^-11^ (0.24)** | **2.72x10^-12^ (-0.39)** |
| rs28362683 | 32480941 | *BTNL2* | 1.50x10^-7^ (0.27) | **1.78x10^-9^ (-0.46)** | 3.49x10^-5^ (0.19) | 1.38x10^-3^ (-0.16) | **4.56x10^-11^ (0.24)** | **1.40x10^-11^ (-0.38)** |
| rs10947261 | 32481210 | *BTNL2* | 1.01x10^-7^ (0.27) | **2.77x10^-10^ (-0.48)** | 2.87x10^-5^ (0.19) | 1.30x10^-3^ (-0.16) | **2.37x10^-11^ (0.25)** | **2.65x10^-12^ (-0.39)** |
| rs10947262 | 32481290 | *BTNL2* | 2.39x10^-7^ (0.26) | **5.38x10^-10^ (-0.46)** | 2.90x10^-5^ (0.19) | 1.26x10^-3^ (-0.16) | **5.41x10^-11^ (0.24)** | **4.38x10^-12^ (-0.38)** |
| rs9268658 | 32518694 | *HLA-DRA* | 4.51x10^-6^ (0.18) | **1.68x10^-8^ (-0.33)** | 7.77x10^-5^ (0.14) | 1.34x10^-3^ (-0.13) | **3.02x10^-9^ (0.17)** | **5.52x10^-11^ (-0.29)** |
| rs8084 | 32519013 | *HLA-DRA* | 1.50x10^-6^ (0.20) | **3.37x10^-9^ (-0.37)** | 1.29x10^-4^ (0.13) | 2.32x10^-3^ (-0.08) | **2.64x10^-9^ (0.18)** | **2.09x10^-10^ (-0.28)** |
| rs2239804 | 32519501 | *HLA-DRA* | 6.20x10^-6^ (0.18) | **2.41x10^-8^ (-0.32)** | 7.77x10^-5^ (0.14) | 1.34x10^-3^ (-0.13) | **4.05x10^-9^ (0.17)** | **7.74x10^-11^ (-0.28)** |
| rs7192 | 32519624 | *HLA-DRA* | 1.71x10^-7^ (0.22) | **3.04x10^-10^ (-0.40)** | 3.04x10^-5^ (0.17) | 1.15x10^-3^ (-0.15) | **6.10x10^-11^ (0.20)** | **4.14x10^-12^ (-0.32)** |
| rs3129888 | 32519704 | *HLA-DRA* | 1.79x10^-5^ (0.22) | **2.53x10^-8^ (-0.43)** | 2.27x10^-4^ (0.13) | 8.20x10^-4^ (-0.20) | **4.32x10^-8^ (0.19)** | **1.08x10^-10^ (-0.36)** |
| rs2239803 | 32519811 | *HLA-DRA* | 4.38x10^-7^ (0.21) | **1.78x10^-9^ (-0.36)** | 4.87x10^-5^ (0.16) | 1.53x10^-3^ (-0.12) | **1.93x10^-10^ (0.19)** | **1.97x10^-11^ (-0.30)** |
| rs2239802 | 32519824 | *HLA-DRA* | 1.10x10^-5^ (0.22) | **1.38x10^-8^ (-0.42)** | 1.13x10^-4^ (0.15) | 8.34x10^-4^ (-0.19) | **3.15x10^-8^ (0.18)** | **2.03x10^-13^ (-0.42)** |
| rs4935356 | 32520366 | *HLA-DRA* | 4.51x10^-6^ (0.18) | **1.68x10^-8^ (-0.33)** | 7.77x10^-5^ (0.14) | 1.34x10^-3^ (-0.13) | **3.02x10^-9^ (0.17)** | **5.52x10^-11^ (-0.29)** |
| rs7194 | 32520458 | *HLA-DRA* | 1.16x10^-7^ (0.22) | **1.96x10^-10^ (-0.40)** | 3.04x10^-5^ (0.17) | 1.15x10^-3^ (-0.15) | **4.27x10^-11^ (0.21)** | **2.81x10^-12^ (-0.33)** |
| rs7195 | 32520517 | *HLA-DRA* | 1.16x10^-7^ (0.22) | **1.96x10^-10^ (-0.40)** | 3.04x10^-5^ (0.17) | 1.15x10^-3^ (-0.15) | **4.27x10^-11^ (0.21)** | **2.81x10^-12^ (-0.33)** |
| rs2213586 | 32521072 | *HLA-DRA* | 1.16x10^-7^ (0.22) | **1.96x10^-10^ (-0.40)** | 3.04x10^-5^ (0.17) | 1.15x10^-3^ (-0.15) | **4.27x10^-11^ (0.21)** | **2.81x10^-12^ (-0.33)** |
| rs2213585 | 32521128 | *HLA-DRA* | 1.16x10^-7^ (0.22) | **1.96x10^-10^ (-0.40)** | 3.04x10^-5^ (0.17) | 1.15x10^-3^ (-0.15) | **4.27x10^-11^ (0.21)** | **2.81x10^-12^ (-0.33)** |
| rs2395182 | 32521295 | *HLA-DRA* | 3.43x10^-6^ (0.23) | **6.32x10^-9^ (-0.43)** | 1.21x10^-4^ (0.15) | 6.86x10^-4^ (-0.21) | **3.41x10^-9^ (0.21)** | **3.46x10^-11^ (-0.36)** |
| rs2227139 | 32521437 | *HLA-DRA* | 1.16x10^-7^ (0.22) | **1.96x10^-10^ (-0.40)** | 3.04x10^-5^ (0.17) | 1.15 x10^-3^ (-0.15) | **4.27x10^-11^ (0.21)** | **2.81x10^-12^ (-0.33)** |
| rs7754768 | 32528157 | *HLA-DRA* | 1.13x10^-7^ (0.22) | **2.20x10^-10^ (-0.40)** | 3.13x10^-5^ (0.17) | 1.00x10^-3^ (-0.16) | **3.82x10^-11^ (0.21)** | **2.99x10^-12^ (-0.32)** |
| rs9268832 | 32535767 | *HLA-DRB9* | **2.25 x10^-8^ (0.24)** | **8.33x10^-11^ (-0.41)** | 8.03x10^-5^ (0.14) | 1.32x10^-3^ (-0.14) | **3.11x10^-11^ (0.21)** | **1.97x10^-12^ (-0.33)** |
| rs9268853 | 32537621 | *HLA-DRB9* | 6.79x10^-8^ (-0.23) | **2.99x10^-8^ (0.33)** | **4.13x10^-8^ (-0.30)** | **1.83x10^-7^ (0.48)** | **3.32x10^-14^ (-0.25)** | **1.32x10^-13^ (0.35)** |
| rs2395185 | 32541145 | *HLA-DRB9* | 1.03x10^-7^ (-0.22) | **4.54x10^-8^ (0.32)** | **4.13x10^-8^ (-0.30)** | **1.83x10^-7^ (0.48)** | **5.30x10^-14^ (-0.25)** | **2.12x10^-13^ (0.35)** |
| rs9368726 | 32546520 | *HLA-DRB5* | 6.79x10^-8^ (-0.23) | **2.99x10^-8^ (0.33)** | **4.13x10^-8^ (-0.30)** | **1.83x10^-7^ (0.48)** | **3.32x10^-14^ (-0.25)** | **1.32x10^-13^ (0.35)** |
| rs9405108 | 32546626 | *HLA-DRB5* | 6.79x10^-8^ (-0.23) | **2.99x10^-8^ (0.33)** | **4.13x10^-8^ (-0.30)** | **1.83x10^-7^ (0.48)** | **3.32x10^-14^ (-0.25)** | **1.32x10^-13^ (0.35)** |
| rs6901541 | 32550239 | *HLA-DRB* | 4.12x10^-7^ (0.23) | **2.49x10^-9^ (-0.40)** | 6.37x10^-5^ (0.16) | 1.51x10^-3^ (-0.13) | **2.77x10^-10^ (0.21)** | **3.83x10^-11^ (-0.32)** |
| rs28366298 | 32668837 | *HLA-DRB1* | **1.39x10^-8^ (-0.25)** | **9.46x10^-9^ (0.36)** | **1.07x10^-7^ (-0.31)** | **1.75x10^-7^ (0.52)** | **1.08x10^-14^ (-0.27)** | **3.87x10^-14^ (0.39)** |
| rs477515 | 32677669 | *HLA-DRB1* | **3.32x10^-9^ (-0.26)** | **5.17x10^-9^ (0.37)** | **5.66x10^-8^ (-0.32)** | **4.73x10^-8^ (0.55)** | **1.37x10^-15^ (-0.28)** | **8.34x10^-15^ (0.40)** |
| rs2516049 | 32678378 | *HLA-DRB1* | **3.32x10^-9^ (-0.26)** | **5.17x10^-9^ (0.37)** | **5.66x10^-8^ (-0.32)** | **4.73x10^-8^ (0.55)** | **1.37x10^-15^ (-0.28)** | **8.34x10^-15^ (0.40)** |
| rs9271488 | 32696978 | *HLA-DQA1* | 1.11x10^-7^ (-0.23) | **4.37x10^-8^ (0.34)** | **7.45x10^-8^ (-0.32)** | **1.37x10^-8^ (0.59)** | **7.54x10^-14^ (-0.26)** | **6.66x10^-14^ (0.38)** |
| rs3104369 | 32710460 | *HLA-DQA1* | 1.89x10^-6^ (0.22) | **1.49x10^-8^ (-0.40)** | 3.75x10^-4^ (0.14) | 2.31x10^-4^ (-0.33) | **7.03x10^-9^ (0.20)** | **1.10x10^-11^ (-0.38)** |

^a^Since we used a liability threshold model for analysis of the dichotomous trait (see methods section), the direction of effect on EBNA-1 discrete serostatus is opposite of the sign of the regression coefficient, but is in the same direction as the regression coefficient for the quantitative trait.
