# Supplementary material for: A Genome-Wide Integrative Genomic Study Localizes Genetic Factors Influencing Antibodies against Epstein-Barr Virus Nuclear Antigen 1 (EBNA-1)
Source: PLoS Genet. 2013 Jan 10;9(1):e1003147. doi: 10.1371/journal.pgen.1003147 (PMC3542101; doi:10.1371/journal.pgen.1003147)
Supplement: Table S3 — Association, conditional on linkage, results for SNP-transcript pairs in the HLA region. Focus is on the 41 SNPs previously found to be significantly associated with EBNA-1 traits (presented in Table 2 and Table 3). Shown are results for the top 74 pairs (p≤1.0×10^−2^). Only four SNP-transcript pairs were significant after adjusting for multiple testing. (DOCX) [file pgen.1003147.s009.docx]

**Table S3.** **Association, conditional on linkage, results for SNP-transcript pairs in the HLA region.** Focus is on the 41 SNPs previously found to be significantly associated with EBNA-1 traits (presented in tables 2 and 3). Shown are results for the top 74 pairs

(*p* ≤ 1.0x10^-2^). Only four SNP-transcript pairs were significant after adjusting for multiple testing.

| Transcript | Gene | SNP | *p*-value (ß_SNP_) |
| --- | --- | --- | --- |
| **GI_14165467-S** | ***RPS18*** | **rs204999** | **4.84x10^-5^** (0.20) |
| **GI_27436886-S** | ***PBX2*** | **rs9273327** | **1.15x10^-4^** (0.28) |
| **GI_27436886-S** | ***PBX2*** | **rs2854275** | **1.67x10^-4^** (0.29) |
| **GI_14165467-S** | ***RPS18*** | **rs10947261** | **2.96x10^-4^** (-0.18) |
| GI_14165467-S | *RPS18* | rs10947262 | 3.72x10^-4^ (-0.18) |
| GI_14165467-S | *RPS18* | rs28362683 | 4.13 x10^-4^ (-0.18) |
| GI_20336252-I | *EGFL8* | rs652888 | 9.85x10^-4^ (0.21) |
| GI_14165467-S | *RPS18* | rs2227139 | 1.02x10^-3^ (-0.14) |
| GI_14165467-S | *RPS18* | rs7195 | 1.02x10^-3^ (-0.14) |
| GI_14165467-S | *RPS18* | rs7194 | 1.02x10^-3^ (-0.14) |
| GI_14165467-S | *RPS18* | rs2213585 | 1.02x10^-3^ (-0.14) |
| GI_14165467-S | *RPS18* | rs2213586 | 1.02x10^-3^ (-0.14) |
| GI_14165467-S | *RPS18* | rs2239803 | 1.07x10^-3^ (-0.13) |
| GI_14165467-S | *RPS18* | rs7192 | 1.09x10^-3^ (-0.14) |
| Hs.427351-S | *REGION-9945* | rs204999 | 1.35x10^-3^ (0.16) |
| GI_27498491-S | *TUBB* | rs10885 | 1.54x10^-3^ (0.23) |
| GI_27498491-S | *TUBB* | rs3130626 | 1.56x10^-3^ (0.23) |
| GI_27498491-S | *TUBB* | rs2736157 | 1.56x10^-3^ (0.23) |
| GI_27498491-S | *TUBB* | rs3115663 | 1.56x10^-3^ (0.23) |
| GI_27498491-S | *TUBB* | rs9267522 | 1.56x10^-3^ (0.23) |
| GI_27498491-S | *TUBB* | rs3130070 | 1.56x10^-3^ (0.23) |
| GI_24475831-S | *NCR3* | rs204999 | 1.60x10^-3^ (-0.13) |
| GI_27894369-S | *NOTCH4* | rs2233956 | 2.16x10^-3^ (-0.26) |
| GI_14574565-A | *AIF1* | rs10885 | 2.61x10^-3^ (-0.22) |
| GI_25777697-A | *TRIM39* | rs3130557 | 2.63x10^-3^ (-0.36) |
| GI_27498491-S | *TUBB* | rs3130623 | 2.67x10^-3^(0.21) |
| GI_24797075-S | *HLA-DPB1* | rs652888 | 3.00x10^-3^ (0.18) |
| GI_18641376-S | *HLA-DMB* | rs3130557 | 3.05x10^-3^ (-0.35) |
| GI_14574565-A | *AIF1* | rs2736157 | 3.46x10^-3^ (-0.21) |
| GI_14574565-A | *AIF1* | rs3130070 | 3.46x10^-3^ (-0.21) |
| GI_14574565-A | *AIF1* | rs9267522 | 3.46x10^-3^ (-0.21) |
| GI_14574565-A | *AIF1* | rs3115663 | 3.46x10^-3^ (-0.21) |
| GI_14574565-A | *AIF1* | rs3130626 | 3.46x10^-3^ (-0.21) |
| GI_6005891-S | *TCF19* | rs10885 | 3.46x10^-3^ (0.21) |
| GI_6005891-S | *TCF19* | rs2736157 | 4.11x10^-3^ (0.21) |
| GI_6005891-S | *TCF19* | rs3115663 | 4.11x10^-3^ (0.21) |
| GI_6005891-S | *TCF19* | rs3130070 | 4.11x10^-3^ (0.21) |
| GI_6005891-S | *TCF19* | rs9267522 | 4.11x10^-3^ (0.21) |
| GI_6005891-S | *TCF19* | rs3130626 | 4.11x10^-3^ (0.21) |
| GI_14165467-S | *RPS18* | rs9268832 | 4.40x10^-3^ (-0.12) |
| GI_18426974-S | *HLA-DQA1* | rs3130557 | 4.54x10^-3^ (0.28) |
| GI_14165467-S | *RPS18* | rs7754768 | 4.66x10^-3^ (-0.12) |
| GI_6005891-S | *TCF19* | rs3130628 | 4.74x10^-3^ (0.20) |
| GI_24797072-S | *HLA-G* | rs2507997 | 5.23x10^-3^ (-0.15) |
| GI_24797072-S | *HLA-G* | rs2854008 | 5.23x10^-3^ (-0.15) |
| GI_11863157-S | *RING1* | rs204999 | 5.65x10^-3^ (-0.14) |
| GI_22091451-S | *APOM* | rs3130623 | 5.68x10^-3^ (0.19) |
| GI_11095446-S | *HLA-DQA2* | rs2233956 | 5.74x10^-3^ (-0.23) |
| GI_20631983-S | *RDBP* | rs2854275 | 6.09x10^-3^ (-0.21) |
| GI_26787973-S | *HSPA1A* | rs2294882 | 6.09x10^-3^ (0.12) |
| GI_26787973-S | *HSPA1A* | rs2294881 | 6.09x10^-3^ (0.12) |
| GI_20631983-S | *RDBP* | rs9273327 | 6.19x10^-3^ (0.19) |
| GI_20357538-A | *ATP6V1G2* | rs3130623 | 6.24x10^-3^ (0.19) |
| GI_34013512-S | *LSM2* | rs3130557 | 6.24x10^-3^ (-0.33) |
| GI_34335278-I | *PSMB8* | rs2233971 | 7.18x10^-3^ (-0.15) |
| GI_22091451-S | *APOM* | rs3130628 | 7.20x10^-3^ (0.19) |
| GI_22091451-S | *APOM* | rs9267522 | 7.33x10^-3^ (0.20) |
| GI_22091451-S | *APOM* | rs3130626 | 7.33x10^-3^ (0.20) |
| GI_22091451-S | *APOM* | rs3130070 | 7.33x10^-3^ (0.20) |
| GI_22091451-S | *APOM* | rs3115663 | 7.33x10^-3^ (0.20) |
| GI_22091451-S | *APOM* | rs2736157 | 7.33x10^-3^ (0.20) |
| GI_26787973-S | *HSPA1A* | rs28362683 | 7.93x10^-3^ (0.14) |
| GI_27498491-S | *TUBB* | rs3130628 | 8.12x10^-3^ (0.19) |
| GI_15451872-S | *B3GALT4* | rs2516049 | 8.26x10^-3^ (0.11) |
| GI_15451872-S | *B3GALT4* | rs477515 | 8.26x10^-3^ (0.11) |
| GI_34335278-A | *PSMB8* | rs2233956 | 8.37x10^-3^ (-0.22) |
| GI_13376368-S | *C6orf134* | rs4248166 | 8.39x10^-3^ (-0.12) |
| GI_22091451-S | *APOM* | rs10885 | 8.83x10^-3^ (0.19) |
| GI_9961245-I | *TAP2* | rs3117583 | 8.87x10^-3^ (-0.18) |
| GI_14574565-A | *AIF1* | rs3130628 | 8.97x10^-3^ (-0.19) |
| GI_20631976-S | *CREBL1* | rs2854008 | 8.98x10^-3^ (-0.14) |
| GI_20631976-S | *CREBL1* | rs2507997 | 8.98x10^-3^ (-0.14) |
| GI_4759179-A | *STK19* | rs9267947 | 9.32x10^-3^ (0.11) |
| GI_10863984-S | *C6orf47* | rs3117583 | 9.36x10^-3^ (0.19) |

Bold=significant at *p* ≤ 3.33x10^-4^ (0.05/150 transcripts)
