# Supplementary material for: A Genome-Wide Integrative Genomic Study Localizes Genetic Factors Influencing Antibodies against Epstein-Barr Virus Nuclear Antigen 1 (EBNA-1)
Source: PLoS Genet. 2013 Jan 10;9(1):e1003147. doi: 10.1371/journal.pgen.1003147 (PMC3542101; doi:10.1371/journal.pgen.1003147)
Supplement: Table S4 — Genetic correlations between HLA transcripts and EBNA-1 serological traits. Only one transcript was significant, after adjusting for multiple testing. (DOCX) [file pgen.1003147.s010.docx]

**Table S4.** **Genetic correlations between HLA transcripts and EBNA-1 serological traits.** Only one transcript was significant, after adjusting for multiple testing.

| Transcript | Gene | Genetic correlation with EBNA-1 quantitative trait (*p*-value) | Genetic correlation with EBNA-1 discrete serostatus trait (*p*-value) |
| --- | --- | --- | --- |
| **GI_18641371-S** | ***HLA-DRB1*** | **0.388 (2.77x10^-5^)** | **0.377 (2.95x10^-4^)** |
| GI_26787990-S | *NFKBIL1* | -0.416 (0.010) | -0.348 (0.061) |
| GI_24797066-S | *HLA-A* | -0.236 (0.013) | -0.178 (0.106) |
| GI_7524353-S | *DDAH2* | -0.480 (0.013) | -0.529 (0.021) |
| GI_10947134-S | *ABCF1* | 0.318 (0.014) | 0.308 (0.032) |
| GI_18375635-S | *BAT4* | -0.512 (0.017) | -0.619 (0.011) |
| Hs.390512-S | *NOTCH4* | 0.900 (0.020) | 0.900 (0.281) |
| GI_24797159-S | *TAP1* | 0.297 (0.029) | 0.363 (0.027) |
| GI_27436892-I | *TAPBP* | 0.355 (0.034) | 0.249 (0.270) |
| GI_26638660-I | *MSH5* | -0.900 (0.035) | -0.900 (0.179) |
| GI_41197088-S | *HLA-DQA1* | 0.196 (0.044) | 0.216 (0.055) |
| GI_32171210-S | *HCG27* | 0.220 (0.051) | 0.218 (0.086) |
| GI_13376252-S | *RPP21* | -0.388 (0.053) | -0.538 (0.019) |
| GI_21237727-S | *DHX16* | 0.379 (0.056) | 0.513 (0.022) |
| GI_23110931-A | *PSMB9* | 0.200 (0.061) | 0.214 (0.071) |
| GI_18201916-I | *COL11A2* | 0.900 (0.062) | 0.900 (0.065) |
| GI_25777697-A | *TRIM39* | 0.900 (0.071) | 0.900 (0.206) |
| hmm31752-S | *REGION-6933* | 0.465 (0.078) | 0.858 (0.008) |
| GI_20631983-S | *RDBP* | 0.395 (0.088) | 0.169 (0.508) |
| GI_42657607-S | *REGION-2328* | 0.184 (0.092) | 0.243 (0.059) |
| GI_34013512-S | *LSM2* | 0.306 (0.101) | 0.332 (0.106) |
| GI_26787963-A | *AGPAT1* | 0.287 (0.106) | 0.157 (0.365) |
| GI_5729965-S | *HCP5* | -0.158 (0.121) | -0.152 (0.216) |
| GI_20631976-S | *CREBL1* | 0.220 (0.123) | 0.165 (0.298) |
| GI_6552331-S | *FLOT1* | 0.180 (0.134) | 0.199 (0.143) |
| GI_18426974-S | *HLA-DQA1* | 0.161 (0.135) | 0.260 (0.033) |
| GI_9961245-I | *TAP2* | 0.193 (0.147) | 0.140 (0.354) |
| GI_15451872-S | *B3GALT4* | -0.177 (0.162) | -0.144 (0.315) |
| GI_26787960-I | *AGER* | -0.388 (0.166) | -0.208 (0.539) |
| GI_14165467-S | *RPS18* | 0.310 (0.173) | 0.108 (0.633) |
| GI_25777705-A | *ZNRD1* | -0.178 (0.187) | -0.175 (0.248) |
| GI_8393383-S | *C6orf48* | -0.169 (0.201) | -0.136 (0.361) |
| GI_18641372-S | *HLA-DRB6* | -0.121 (0.207) | -0.183 (0.099) |
| GI_9665231-S | *HLA-F* | -0.138 (0.210) | -0.094 (0.470) |
| GI_39725662-S | *WDR46* | -0.388 (0.214) | -0.317 (0.376) |
| GI_11497611-A | *GABBR1* | -0.164 (0.222) | -0.056 (0.706) |
| GI_11545816-S | *GPSM3* | -0.554 (0.222) | -0.436 (0.590) |
| GI_16554596-I | *IER3* | 0.182 (0.222) | 0.143 (0.389) |
| GI_34305290-S | *RNF5* | -0.488 (0.224) | -0.033 (0.942) |
| GI_7662666-S | *C6orf15* | 0.900 (0.229) | 0.225 (0.887) |
| Hs.485087-S | *REGION-10973* | 0.900 (0.246) | 0.429 (0.766) |
| GI_25777670-S | *PPP1R10* | -0.241 (0.255) | -0.307 (0.196) |
| GI_34222289-S | *GTF2H4* | -0.186 (0.256) | -0.014 (0.919) |
| GI_18765714-S | *HLA-DMA* | -0.151 (0.257) | -0.106 (0.470) |
| Hs.485086-S | *REGION-10972* | 0.353 (0.272) | -0.182 (0.656) |
| GI_16445440-S | *TRIM26* | 0.302 (0.275) | 0.361 (0.263) |
| GI_11863157-S | *RING1* | -0.201 (0.276) | -0.240 (0.254) |
| GI_22091451-S | *APOM* | -0.900 (0.285) | -0.900 (0.127) |
| GI_9506770-S | *HCG4P10* | 0.254 (0.286) | 0.184 (0.484) |
| GI_38788318-S | *GNL1* | -0.900 (0.288) | -0.900 (0.069) |
| hmm14535-S | *REGION-2836* | -0.296 (0.292) | -0.294 (0.331) |
| GI_18375629-A | *BAT3* | 0.196 (0.295) | 0.318 (0.115) |
| GI_4504410-S | *HLA-DRB1* | -0.099 (0.296) | -0.067 (0.551) |
| GI_20665033-A | *TNXB* | 0.141 (0.298) | 0.087 (0.577) |
| GI_26787971-S | *CSNK2B* | -0.158 (0.298) | -0.172 (0.309) |
| GI_26787973-S | *HSPA1A* | -0.122 (0.315) | -0.166 (0.224) |
| GI_14574567-I | *AIF1* | -0.108 (0.320) | -0.101 (0.422) |
| GI_38455408-S | *PFDN6* | 0.243 (0.320) | 0.057 (0.837) |
| GI_21450766-S | *C6orf136* | 0.180 (0.330) | 0.283 (0.202) |
| GI_24308194-S | *VARS2* | 0.122 (0.334) | -0.055 (0.700) |
| Hs.259036-S | *REGION-9236* | -0.900 (0.339) | 0.056 (0.970) |
| GI_6005891-S | *TCF19* | 0.900 (0.346) | 0.900 (0.587) |
| GI_24797075-S | *HLA-DPB1* | -0.127 (0.355) | 0.109 (0.474) |
| Hs.519979-S | *HLA-K* | -1.000 (0.355) | -0.900 (0.157) |
| GI_11386174-A | *PPP1R11* | -0.230 (0.376) | -0.129 (0.678) |
| GI_6680470-I | *AIF1* | -1.000 (0.379) | -0.900 (0.227) |
| GI_18379339-A | *VPS52* | -0.219 (0.397) | -0.252 (0.407) |
| GI_27436894-I | *TAPBP* | -0.258 (0.397) | -0.315 (0.438) |
| GI_27894369-S | *NOTCH4* | 1.000 (0.397) | 0.900 (0.576) |
| GI_10863984-S | *C6orf47* | -0.442 (0.398) | -0.594 (0.431) |
| GI_6996015-A | *LTB* | 0.167 (0.399) | 0.140 (0.539) |
| GI_21327676-S | *HLA-B* | 0.088 (0.404) | 0.251 (0.042) |
| GI_20336250-A | *PPT2* | 0.900 (0.411) | -0.135 (0.906) |
| GI_24797072-S | *HLA-G* | 0.176 (0.415) | 0.178 (0.442) |
| GI_16519560-I | *TRIM10* | 0.125 (0.423) | 0.116 (0.501) |
| GI_20070223-S | *ZBTB22* | 0.161 (0.427) | 0.151 (0.503) |
| GI_11095446-S | *HLA-DQA2* | -0.117 (0.442) | -0.165 (0.342) |
| GI_5901935-S | *SLC39A7* | -0.900 (0.453) | 0.078 (1.000) |
| GI_26787964-I | *AGPAT1* | 0.901 (0.464) | -0.035 (1.000) |
| Hs.443566-S | *REGION-10174* | 0.231 (0.484) | 0.121 (0.746) |
| GI_27436942-S | *RXRB* | -0.416 (0.487) | -0.420 (0.442) |
| GI_18641378-S | *HLA-DRA* | 0.086 (0.489) | 0.050 (0.748) |
| GI_25282390-S | *NRM* | -0.128 (0.489) | -0.128 (0.847) |
| GI_18426878-A | *EHMT2* | -1.000 (0.503) | -0.900 (0.149) |
| GI_40538792-S | *CCHCR1* | -0.105 (0.510) | 0.089 (0.628) |
| GI_13376875-S | *LY6G5C* | 0.169 (0.520) | 0.184 (0.497) |
| GI_45580711-A | *BAT1* | 0.088 (0.525) | 0.305 (0.072) |
| GI_26787987-S | *MICB* | -0.083 (0.526) | 0.007 (0.939) |
| GI_40806202-S | *NEU1* | -0.131 (0.527) | -0.251 (0.267) |
| GI_38327631-A | *DDR1* | -0.126 (0.533) | -0.203 (0.376) |
| GI_20143980-S | *HSD17B8* | 0.110 (0.545) | 0.150 (0.440) |
| hmm31743-S | *REGION-6927* | -0.083 (0.554) | -0.170 (0.277) |
| GI_16554595-A | *IER3* | 0.075 (0.561) | 0.150 (0.290) |
| GI_18379336-I | *VPS52* | -0.152 (0.562) | -0.069 (0.801) |
| GI_12408641-S | *BRD2* | 0.110 (0.570) | 0.273 (0.217) |
| Hs.132807-S | *3.8-1* | -0.462 (0.577) | -0.412 (0.606) |
| GI_27498491-S | *TUBB* | 0.071 (0.604) | 0.086 (0.576) |
| Hs.398075-S | *REGION-9836* | -0.914 (0.624) | -0.900 (0.397) |
| GI_14589873-A | *DOM3Z* | 0.153 (0.630) | 0.310 (0.363) |
| GI_7662664-S | *PSORS1C2* | -0.904 (0.635) | -0.900 (0.275) |
| GI_13376368-S | *C6orf134* | -0.911 (0.644) | 0.004 (1.000) |
| GI_27436896-A | *TAPBP* | -0.425 (0.647) | -0.077 (1.000) |
| GI_20336252-I | *EGFL8* | -0.100 (0.653) | 0.024 (0.891) |
| GI_9961247-I | *TAP2* | -0.107 (0.660) | -0.393 (0.188) |
| GI_19557676-S | *HLA-C* | 0.042 (0.671) | 0.087 (0.438) |
| GI_16554601-S | *MRPS18B* | 0.080 (0.678) | -0.039 (0.875) |
| GI_7661965-S | *MDC1* | 0.045 (0.678) | -0.006 (0.941) |
| GI_13376867-A | *SLC44A4* | -0.701 (0.684) | 0.048 (0.925) |
| GI_4557750-S | *REGION-2762* | -0.038 (0.684) | -0.100 (0.347) |
| GI_25777706-I | *ZNRD1* | -0.079 (0.694) | 0.027 (0.904) |
| GI_26638665-A | *C6orf26* | -0.184 (0.709) | -0.014 (1.000) |
| GI_31542491-S | *DAXX* | -0.070 (0.720) | 0.007 (0.976) |
| GI_26787974-S | *HSPA1B* | 0.042 (0.722) | 0.067 (0.609) |
| GI_14251208-S | *CLIC1* | 0.070 (0.726) | -0.197 (0.391) |
| GI_34335278-I | *PSMB8* | 0.064 (0.733) | 0.214 (0.305) |
| GI_20357538-A | *ATP6V1G2* | -0.069 (0.734) | -0.151 (0.504) |
| GI_34304364-S | *FKBPL* | 0.902 (0.735) | 0.900 (0.349) |
| GI_24797068-S | *HLA-DQB1* | 0.033 (0.738) | 0.042 (0.714) |
| GI_4759179-A | *STK19* | -0.076 (0.741) | 0.143 (0.575) |
| GI_34335277-I | *PSMB8* | 0.902 (0.748) | 0.900 (0.102) |
| GI_14577918-S | *C4B* | 0.188 (0.752) | -0.198 (0.792) |
| GI_14249467-I | *SLC44A4* | -0.078 (0.771) | -0.209 (0.475) |
| GI_30160064-S | *REGION-1116* | 0.062 (0.777) | 0.204 (0.427) |
| GI_20631970-S | *C2* | -0.066 (0.786) | -0.053 (0.845) |
| GI_24475831-S | *NCR3* | 0.025 (0.786) | -0.074 (0.491) |
| Hs.427351-S | *REGION-9945* | 0.901 (0.787) | 0.082 (1.000) |
| GI_26787961-A | *AGER* | 0.036 (0.813) | 0.034 (0.849) |
| GI_27436886-S | *PBX2* | -0.071 (0.821) | -0.111 (0.746) |
| GI_34419636-S | *HLA-DOA* | 0.087 (0.829) | 0.006 (1.000) |
| GI_37552135-S | *KIFC1* | -0.098 (0.837) | -0.256 (0.615) |
| GI_16445353-A | *TRIM31* | -0.900 (0.847) | -0.900 (0.475) |
| GI_19913380-A | *C6orf25* | 0.079 (0.849) | 0.187 (0.723) |
| GI_20555643-S | *KIAA1949* | -0.039 (0.853) | -0.005 (1.000) |
| GI_18641376-S | *HLA-DMB* | 0.030 (0.857) | 0.142 (0.444) |
| GI_15100150-S | *BAT5* | 0.900 (0.863) | -0.050 (1.000) |
| GI_20631986-S | *SKIV2L* | 0.021 (0.883) | -0.037 (0.823) |
| GI_25952110-S | *TNF* | 0.018 (0.886) | 0.123 (0.397) |
| GI_24797070-S | *HLA-E* | -0.015 (0.900) | 0.016 (0.860) |
| GI_34335278-A | *PSMB8* | -0.020 (0.905) | -0.050 (0.783) |
| GI_27483210-S | *REGION-532* | -0.013 (0.907) | -0.117 (0.345) |
| GI_21361071-S | *RGL2* | -0.032 (0.912) | 0.083 (0.758) |
| GI_27436928-S | *HSPA1L* | 0.015 (0.913) | -0.048 (0.753) |
| GI_5454157-S | *VARS* | -0.013 (0.950) | -0.142 (0.534) |
| GI_6806892-S | *LTA* | -0.008 (0.958) | 0.059 (0.740) |
| GI_20631979-I | *DOM3Z* | -0.010 (0.971) | -0.105 (0.753) |
| GI_20555690-S | *REGION-247* | 0.005 (0.973) | 0.058 (0.701) |
| GI_14574565-A | *AIF1* | -0.004 (0.977) | 0.034 (0.865) |
| GI_13376877-S | *PRR3* | -0.004 (0.984) | -0.062 (0.767) |
| GI_14574565-I | *AIF1* | -0.003 (0.991) | 0.281 (0.355) |

Bold=significant at *p* ≤ 3.33x10^-4^ (0.05/150 transcripts)
